# Pet and owner personality and mental wellbeing associate with attachment to cats and dogs

**DOI:** 10.1016/j.isci.2023.108423

**Published:** 2023-11-09

**Authors:** Aada Ståhl, Milla Salonen, Emma Hakanen, Salla Mikkola, Sini Sulkama, Jari Lahti, Hannes Lohi

**Affiliations:** 1 Department of Psychology and Logopedics, University of Helsinki, 00014 Helsinki, Finland; 2 Department of Veterinary Biosciences, University of Helsinki, 00014 Helsinki, Finland; 3 Department of Medical and Clinical Genetics, University of Helsinki, 00014 Helsinki, Finland; 4 Folkhälsan Research Center, 00290 Helsinki, Finland; 5 Department of Biology, University of Turku, 20014 Turku, Finland

**Keywords:** Anthrozoology, Canine behavior, Feline behavior, Psychology

## Abstract

Human-pet attachment can impact the life of both parties, and the identification of underlying characteristics related to attachment style can improve human-pet relationships. We employed structural equation modeling (SEM) to explore associations between human, dog, and cat personalities, owner mental well-being, unwanted pet behavior, and attachment styles in a sample of 2,724 Finnish pet owners (92% women) and their 2,545 dogs and 788 cats. Our findings reveal that owner neuroticism and poor mental well-being are linked to anxious pet attachment in both dog and cat owners. Pet characteristics, such as unwanted behavior and lower human sociability are associated with avoidant attachment style. Overall, this study highlights the significance of individual traits in both pets and owners contributing to insecure attachment styles and underscores the potential to enhance the well-being of both pets and their owners through a deeper understanding of these traits.

## Highlights

• Anxiously attached owners scored lower in mental wellbeing and higher in neuroticism
• Cats' activity and dogs' fear-behavior were linked with higher attachment anxiety
• Attachment avoidance was associated with aggression and ADHD-like behavior in dogs
• Human sociability was associated with lower attachment avoidance in both pet species

## Graphical abstract

**Figure:**
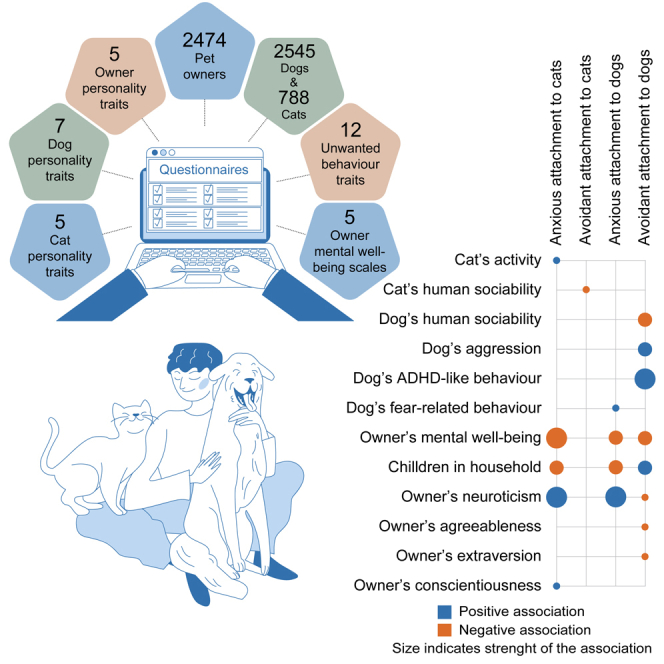

## Introduction

The strong attachment bond between humans and their pets increases the welfare of both parties,^1^^,^^2^^,^^3^^,^^4^^,^^5^ but when compromised, it may increase the owner’s stress and weaken the quality of life.^2^^,^^6^ The attachment framework has been utilized to explain the human-dog and human-cat relationship.^2^^,^^7^^,^^8^^,^^9^^,^^10^^,^^11^ Zilcha-Mano et al.^2^ applied the two-dimensional model of adult attachment^12^ to examine the human-pet bond, in which people are considered to differ in their attachment to pets along the two dimensions of insecurity: (1) attachment-related anxiety, i.e., tendency to be concerned that something bad might happen to the pet and need for a high level of proximity, and (2) attachment-related avoidance, i.e., need for physical and emotional distance from the pet as well as difficulties in seeking support from the pet.^2^

Previous studies indicate that in pet-owner relationships, attachment style may be related to psychological well-being and life quality of the owner,^2^^,^^6^ amount of care and attention the pet receives,^5^^,^^6^ and problem behavior of the pet.^13^^,^^14^^,^^15^^,^^16^^,^^17^ Insecure attachment style may even lead to relinquishment of dogs.^18^ Personality traits of pets have been associated with reported relationship satisfaction and strength of the emotional bond^14^^,^^19^^,^^20^^,^^21^^,^^22^^,^^23^^,^^24^^,^^25^^,^ but to our knowledge, the relationship between pet personality and owner’s attachment insecurity has not yet been studied.

Several researchers have sought to understand how the Big Five traits (neuroticism, the tendency to experience negative emotions^26^; extraversion, the tendency to be active, desire for social attention and exhibit positive affect^27^; openness to experiences, the appreciation for imagination and curiosity^28^; agreeableness, the tendency to maintain social harmony and be selfless^29^; and conscientiousness, the tendency to be self-disciplined and cautious^30^) relate to adult attachment insecurity. Higher neuroticism and lower conscientiousness have been linked to attachment anxiety in adult relationship, lower extraversion and lower conscientiousness have been associated with avoidant attachment, and lower agreeableness has been linked to both avoidant and anxious attachment.^31^ Recent studies have further suggested that high neuroticism, low conscientiousness, low extraversion, and low openness also link to pet attachment insecurity^2^^,^^32^^,^^33^ of the owner-pet relationship, as well as the quality of the relationship and affection toward the pet.^19^^,^^20^^,^^32^ However, not all studies have found any associations between owner personality dimensions and relationship quality.^3^^,^^22^^,^^25^

The quality of the human-pet attachment can substantially impact the lives of many people and their pets. The initial step in improving the owner-pet relationship is to identify factors that determine the attachment bond. This study represents the first cross-species examination of how individual traits from both sides of the relationship are associated with human-pet attachment insecurity. More specifically, we examine the associations of owner, dog, and cat personality traits, as well as unwanted behavior of pets, with owner attachment anxiety and avoidance.

## Results

We collected our dataset using an owner personality and well-being survey, which measured owner personality, attachment to pets with the Pet Attachment Questionnaire (PAQ)^2^ and mental well-being, including, for example, satisfaction with life and perceived stress. We combined these data with data collected from these owners’ dogs and cats with validated personality and behavioral questionnaires.^34^^,^^35^ We used structural equation modeling (SEM) to examine the association of owner personality and well-being and pet personality and unwanted behavior with attachment insecurity. We defined eight competing structures which differed by type of attachment scores (original scales/latent variables), the number of owner personality traits included (based on previous literature^2^^,^^31^^,^^32^^,^^33^), and type of latent well-being variable (single latent variable/two latent variables; Figure S1). We selected the final structures for dog and cat owners by comparing the model fit of these competing structures.

### Study cohort

The data included 2,724 Finnish pet owners (of which 2,150 were dog owners, 540 were cat owners, and 34 owned both dogs and cats) who filled out the survey of 2,545 dogs and 788 cats. These owners had filled at least one survey module of themselves and one of their animals. Of all the pet owners, 2,508 of the pet owners identified as women, 79 as men, 28 as other or did not want to tell, and for 109 gender was unknown, and the modal age group was 25–29 years. Descriptive statistics are presented in Tables S1–S3. See Table S1 for categories, their frequencies, and proportion of missing data for all categorical variables and Tables S2 and S3 for ranges, means, standard deviations, and proportion of missing data for continuous variables.

### Model comparison and fit

Overall, the first model reached best model fit for cat owners and dog owners (Table S4). In this model, well-being scales (Satisfaction with Life Scale, SWLS^36^; Perceived Stress Scale, PSS^37^; Warwich-Edinburgh Mental Well-being Scale, WEMWBS^38^; Generalized Anxiety Disorder Scale, GAD-7^39^; and Center of Epidemiologic Studies Depression Scale, CESD-10^40^) reduced into one latent variable we named total well-being, as scales in which higher scores indicate better well-being loaded positively and scales in which higher scores indicate poorer well-being loaded negatively. This model also included attachment styles as scales instead of latent variables and a more comprehensive set of owner personality traits.

In the final model of dog owners, absolute fit indices indicated good model fit: RMSEA (Root-Mean-Square Error of Approximation; lower RMSEA values [typically below 0.08] indicate better fit^41^) = 0.054 and SRMR (Standardized Root-Mean-Square Residual; lower SRMR values [typically below 0.08] suggest better model fit^41^) = 0.067. Relative fit indices indicated poor fit: CFI (Comparative Fit Index; values closer to 1 indicate better fit^41^) = 0.880 and TLI (Tucker-Lewis Index; values closer to 1 indicate better fit^41^) = 0.854. However, the RMSEA of the null model was 0.140, and therefore relative fit indices may not be informative.^41^

In the final model of cat owners, absolute fit indices indicated good model fit: RMSEA = 0.060 and SRMR = 0.060. Relative fit indices indicated poor fit: CFI = 0.880 and TLI = 0.842. However, the RMSEA of the null model was 0.152, and therefore relative fit indices may not be informative for the cat owner model either.

### Factors associated with attachment avoidance

**Figure 1:**
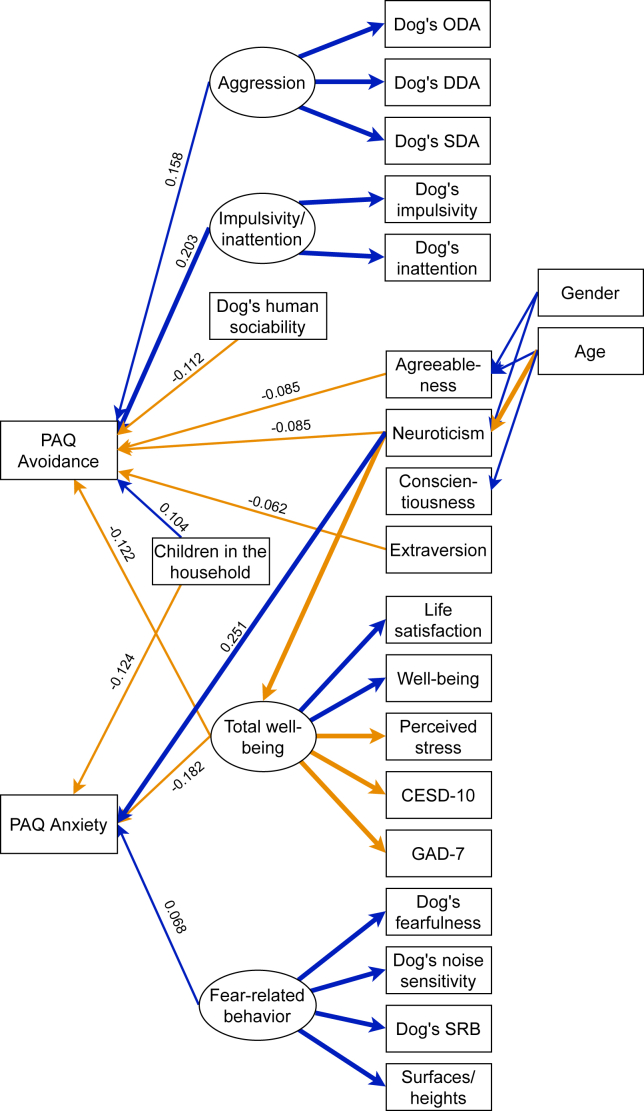
SEM structure of attachment insecurity in dog owners Significant (p < 0.05) standardized estimates are included and paths over 0.2 and under −0.2 are bolded. Positive paths are blue and negative paths orange. Covariances, variances, and intercepts are omitted for clarity. All associations can be found in Table S9. PAQ, Pet Attachment Questionnaire; ODA, owner-directed aggression; DDA, dog-directed aggression; SDA, stranger-directed aggression; CESD-10, Center of Epidemiologic Studies Depression Scale; GAD-7, Generalized Anxiety Disorder Scale; SRB, separation-related behavior, and surfaces/heights = fear of surfaces/heights.

**Figure 2:**
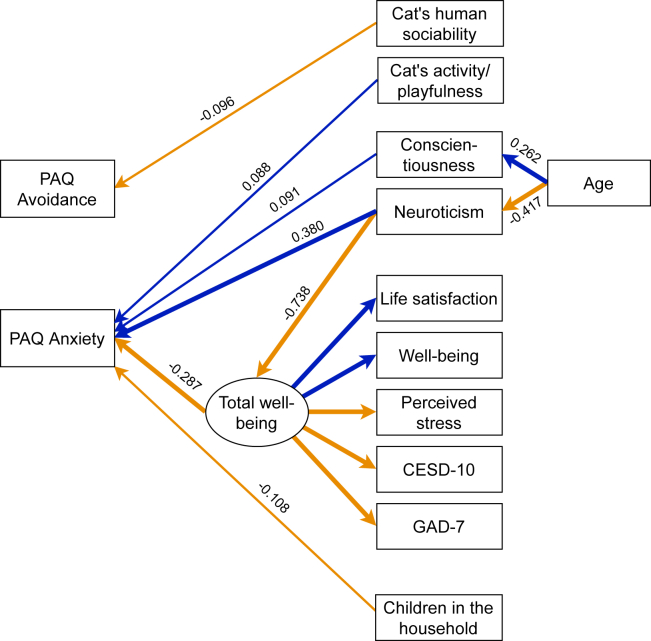
SEM structure of attachment insecurity in cat owners Significant (p < 0.05) standardized estimates are included and paths over 0.2 and under −0.2 are bolded. Positive paths are blue and negative paths orange. Covariances, variances, and intercepts are omitted for clarity. All associations can be found in Table S10. PAQ, Pet Attachment Questionnaire; CESD-10, Center of Epidemiologic Studies Depression Scale; GAD-7, Generalized Anxiety Disorder Scale.

More avoidantly attached dog owners had lower scores in agreeableness, neuroticism, and extraversion and lower scores in total well-being. High attachment avoidance score was also associated with lower scores in human sociability, and higher scores in impulsivity/inattention and aggression in dogs (Figure 1; Table S5). Dog owners with children scored higher on attachment avoidance than those without children (Table S5), as did dog owners who were not female. Owners of cats with lower scores in human sociability had higher scores in avoidant attachment (Figure 2; Table S6).

### Factors associated with attachment anxiety

Dog owners with higher scores in neuroticism and lower scores in total well-being had higher scores in anxious attachment. High score in fear-related behavior of the dog was also associated with higher attachment anxiety of owners (Figure 1; Table S5). Dog owners who had children had lower scores in anxious attachment than those who did not have children (Table S5).

Cat owners with higher scores in neuroticism and conscientiousness and lower scores in total well-being had higher scores in anxious attachment. Cat owners with more active/playful cats had also higher scores in anxious attachment (Figure 2; Table S6). Cat owners with children had lower scores in anxious attachment than those without children.

All results are found in Tables S5–S12.

## Discussion

Our large cross-species study revealed the characteristics of humans and their pet companions that related to insecure human-pet attachment. The results of this study indicated that individual traits of both owners and their pets are associated with attachment insecurity. To our knowledge, this is the first study to look at the role of personality on both sides of the attachment relationship, and the first to look at both the human-dog and the human-cat bond separately. A separate examination of the relationship with the two pet species allowed for a more detailed investigation of the unique associations of personality traits in human-dog and human-cat attachment. A relatively big sample size allowed the utilization of a uniquely large number of variables within the SEM framework. In addition, the strength of this study is the measurement of dog and cat personalities by validated surveys in which the personality structure has been discovered based on extensive data (more than 15,300 dogs and more than 4,300 cats). Moreover, unlike many previous studies examining the relationship between the human and the pet, we used a validated method to assess the quality of the relationship. However, due to the gender bias and the primarily Finnish sample, caution is needed when applying these findings to pet owners of different genders or other cultural contexts.

This study follows previous findings of Reevy and Delgado^32^^,^^33^ and Zilcha-Mano et al.^2^ indicating that cat and dog owners with higher neuroticism are more anxiously attached to their pets. This is also in line with studies of attachment in relationships between adults.^31^ Hence, neuroticism seems to be related to anxious attachment regardless of the attachment figure. Neuroticism is characterized by negative emotionality reflecting insecurity, anxiety, and threat detection,^42^ which may explain this association given that attachment anxiety reflects sensitivity to experiencing negative emotions in the context of the relationship. While neuroticism predisposes one to conflicts and detrimental behaviors in human relationships,^43^ Reevy and Delgado^32^ pointed out that anxious attachment may, on the contrary, have benefits for the pet because anxiously attached owners may be more alert and sensitive to changes in their pet’s behavior and health. Attachment anxiety has been linked to higher affection toward the pet^32^ and providing more caregiving behavior and attention to the pet.^4^^,^^5^ However, attachment anxiety may be distressing for the owner as it has been linked to lower psychological well-being.^2^^,^^6^ Supporting this notion, anxiously attached pet owners scored lower in total well-being in this study as well. Interestingly, despite a strong negative association between neuroticism and total well-being, poor well-being was still independently associated with higher attachment anxiety.

Other personality traits of the owner were also associated with attachment insecurity. Based on previous studies, we expected that extraversion^2^^,^^32^ and conscientiousness^32^^,^^33^ would associate with lower avoidant attachment to cats and dogs. Indeed, we found that higher extraversion in the owners associated with lower avoidant attachment with the dogs but not with the cats, while conscientiousness was not associated with avoidant attachment. A previous study discovered an association between high neuroticism and low attachment avoidance in cats,^32^^,^^33^ which we replicated for dogs but not for cats. Furthermore, we did not find a previously discovered association between avoidant attachment and openness.^30^ We also expected that more conscientious cat owners would be less anxiously attached,^33^ but discovered that more conscientious cat owners were, in contrast, more anxiously attached. We also discovered that agreeableness was associated with lower avoidant attachment in dog owners. Although this has not been found in previous studies of pet attachment, this is in line with previous research regarding related concepts that have found a link between agreeableness and positive aspects of the human-pet relationship; stronger human-dog bond^19^ and relationship quality.^20^ A connection between agreeableness and avoidant attachment has also been found in studies of human relationships.^31^

Human sociability of dogs and cats was associated with lower avoidant attachment in this study. In other words, the pet’s own tendency to seek closeness and interaction in the relationship was associated with a similar tendency in the owner. Human sociability in dogs has been previously linked to more positive owner-perceived attributions of the dog.^44^ It has been speculated that the characteristics of a cat, e.g., independence, could explain the tendency of cat owners to have a greater avoidant attachment.^2^ The human-pet attachment may benefit from higher sociability of the pet, or it could be that less avoidantly attached owners assess their pets as more social, select pets that are more social or behave in a way that affects the pet’s sociability.

Activity/playfulness in cats was associated with higher attachment anxiety in pet owners. To our knowledge, the association of these personality traits with attachment insecurity has not been studied. High activity level of a cat could elicit feelings of inadequacy in owners, if these cats do not seem content despite extensive environmental enrichment.

Unwanted behavior of dogs was associated with attachment insecurity. Based on previous studies, we expected that aggression of dogs^13^ would be linked to avoidant attachment, and the current results add support for this notion. Gobbo and Zupan^13^ proposed that more avoidantly attached owners may not provide enough security in threatening situations, provoking fear, and aggressive behavior. In addition, dog’s impulsivity was associated with higher avoidant attachment. More avoidantly attached owners may participate less in shared activities with the dog, which is related to the dog’s impulsiveness,^45^ devote less time to training, or perceive their dogs as more disobedient. Impulsivity/inattention is also highly negatively associated with training focus^46^ which has been previously linked to a stronger emotional bond.^14^^,^^24^ It is also possible that the dog’s impulsivity and aggression may negatively affect the attachment bond. As a novel finding, we also discovered that fear-related behavior of dogs was associated with higher attachment anxiety in their owners. Fearfulness of the dog could evoke worry for the dog’s wellbeing, but it is also possible that the owner’s anxious attachment leads to anxious behavior of the dog.

In our study, we did not explicitly delve into the direction of causation or the extent of interdependence between the personality traits of humans and their pets, which would require longitudinal research. Personality traits are influenced by both genetic factors and environmental experiences^47^ and relatively stable, and this stability tends to increase with age.^48^ As our participants were predominantly adults, the personality traits of pet owners are likely relatively independent of those exhibited by their pets. On the other hand, most pets are obtained during the sensitive period and thus, the consistent influence of their owners may shape their personalities. Furthermore, attachment theory assumes a bidirectional relationship influenced by both socialization (environment to person) and selection (person to environment) effects,^49^ meaning that current and past attachment experiences and personality of both parties may shape attachment. Therefore, future studies could examine these potential causal directions.

In conclusion, traits of pet owners, more specifically poor well-being and neurotic personality, are linked to the anxious pet attachment style. In turn, the pet’s personality and behavior, such as lower human sociability and unwanted behavior, seem to be related to the avoidant attachment style. Human sociability appeared to be a favorable trait of both pet species regarding attachment avoidance. These results may have practical implications for enhancing human-pet relationships. For example, recognizing the link between owner neuroticism and poor mental well-being with anxious pet attachment enables pet owners to become more conscious of their emotional responses and potentially seek ways to offer greater comfort and stability to their pets. Additionally, when pet owners seek assistance for their pet’s behavioral issues, it may be possible to enhance interventions by also considering the owner’s attachment style. Furthermore, a deeper understanding of these connections can assist prospective pet owners in making more informed decisions when acquiring a pet. It is crucial to acknowledge that obtaining a pet while experiencing poor mental well-being may not necessarily meet expectations of improving one’s psychological health. Instead, acknowledging the potential risk of an insecure attachment is crucial, as it could potentially harm the well-being of both the pet and the owner.

Future research holds exciting prospects for investigating the causal mechanisms behind the associations we have observed. Potential differences in the training methods of avoidantly and anxiously attached pet owners and associated factors to secure attachment style (when low levels are observed in both dimensions) would be exciting topics for future research. In addition, complementarity between pet owners and their pets has been previously linked to higher relationship satisfaction.^3^^,^^25^^,^^50^^,^^51^ In this regard, investigating if the compatibility of owner and pet personalities affects the attachment insecurity is another interesting direction for future research. Overall, these findings suggested that the human-pet relationship not only has links to human personality but also to the personality and behavior of the pet. Future research is needed to clarify these associations further.

### Limitations of the study

This study has limitations. Directionality or causal relationships between personality traits and attachment cannot be derived due to the cross-sectional design of this study. However, these results provide an initial understanding of how cross-species personality may be related to attachment, and future studies should aim to study the causal mechanisms underlying the associations. The second limitation is the limited diversity of the sample, as participants predominantly identified as female and were of Finnish origin. Previous studies have indicated gender differences in how an individual is attached to one’s pet,^32^^,^^33^^,^^52^^,^^53^^,^^54^^,^^55^ so future studies should use more heterogeneous samples to explore the generalisability of our findings. Additionally, we did not consider the potential influence of the sex of the pets, despite prior research suggesting its role in shaping relationship structures.^56^ The analyses were based on questionnaires, and participation requires effort and therefore, the data might be biased toward more enthusiastic and engaged pet owners. Pet owners scoring high on the attachment insecurity dimensions could experience their pets in a less favorable manner reflecting their internal working model of others and not the objective behavior of their pets. In future studies, it would be interesting to examine if more avoidantly or anxiously attached owners’ assessment of their pets’ personality differs from that of an experienced external evaluator. However, by using questionnaires our study is comparable to earlier studies linking pet personality with attachment as they have also used reports of the owners. We were also interested in examining owners owning both cats and dogs, but we could not do that due to the small sample size of multi-species owners (N = 34). In future, it would be interesting to compare the results of multi-species owners to owners owning only cats or dogs.

## STAR★Methods

### Key resources table

**Table:** 

REAGENT or RESOURCE	SOURCE	IDENTIFIER
**Deposited data**		

Data used in this study	Corresponding author	Survey data used in this study is available from the corresponding author upon request.

**Software and algorithms**		

Code used in this study	Supplemental information	Data S1
R 4.2.1.	R Core Team	https://cran.r-project.org/

### Resource availability

#### Lead contact

Further information and requests for resources should be directed to and will be fulfilled by the lead contact, Hannes Lohi (hannes.lohi@helsinki.fi).

#### Materials availability

This study did not generate new unique materials.

#### Data and code availability

• Questionnaire data are available from the corresponding author on reasonable request.
• All original code is available in this paper’s supplemental information.
• Any additional information required to reanalyse the data reported in this paper is available from the lead contact upon request.

### Experimental model and study participant details

Pet owners participated in the study by answering an extensive online behavior questionnaire of their dogs or cats and personality and well-being questionnaire of themselves. Range, mean, and standard deviation of pet owners’ age and gender identity can be found in. Of all the pet owners, 2 508 of the pet owners identified as women, 79 as men, 28 as other or did not want to tell, and for 109 gender was unknown, and the modal age group was 25–29 years. The associations of age and gender are found in Tables S5 and S6. Pet owners provided the information of their pet’s sex, age, breed, environment, and personality. We have approval from the University of Helsinki Viikki Campus (Animal) Research Ethics Committee for the pet behavior surveys (2/2019). For collecting personality and welfare information of the pet owners, we have approval from The University of Helsinki Ethical Review Board in Humanities and Social and Behavioral Sciences (29/2020). Informed consent was obtained from all participants.

### Method details

#### Procedure

The data were collected from November 2018 to May 2021 through online surveys. Participants were recruited through an advertisement posted on social media. The owner personality and well-being survey consisted of three modules: personality, pet attachment, and well-being. In addition, the participants were asked to fill out a validated personality and behavioral questionnaire^34^^,^^35^ of their dog or cat that consisted of background, health, and behavior sections.

In the owner personality module, participants filled out the 64-item Short Five Inventory (S5).^57^ The S5 has shown good psychometric properties in earlier studies.^57^^,^^58^ Internal consistency ranged from α = 0.65 (agreeableness) to α = 0.86 (neuroticism) in our study. In the attachment section, participants were given the 26-item Pet Attachment Questionnaire (PAQ)^2^ relating to owner-perceived attachment insecurity. The PAQ has reported high levels of test-retest reliability (0.75 for the anxiety scale and 0.80 for the avoidance scale).^2^ In the current study, internal consistency ranged from α = 0.83 for pet attachment anxiety to α = 0.79 for pet attachment avoidance, and a nonsignificant correlation between the two scales, r = 0.04. In the well-being section, participants filled out the 5-item Satisfaction with Life Scale (SWLS),^36^ the 10-item Perceived Stress Scale (PSS),^37^ the 7-item Warwich-Edinburgh Mental Well-being Scale (WEMWBS),^38^ the 7-item Generalized Anxiety Disorder Scale (GAD-7),^39^ and the 10-item Center of Epidemiologic Studies Depression Scale (CESD-10).^40^ We also collected background information about age, gender, and children in the household. For missing data in individual items, we used person mean imputation for each scale utilizing the package *TestDataImputation*.^59^

#### Dog personality and behavior

To collect behavioral, personality and background information from dogs, we used the validated dog personality and unwanted behavior questionnaire.^35^ Based on a dataset of over 15 300 responses, Salonen et al.^35^ previously reduced the items of the behavior sections based on reliability, missingness and item loadings utilizing factor analysis and found that the items tap into seven personality traits and nine unwanted behavior traits. The personality factors comprise of insecurity, training focus, energy, aggressiveness/dominance, human sociability, dog sociability and perseverance. The unwanted behavior sections tap into factors of noise sensitivity, fearfulness, aggression toward strangers, aggression toward the owner, aggression toward dogs, fear of surfaces and heights, separation anxiety, hyperactivity/impulsivity, and inattention. See Table S13 for sample items. The items and the factors of impulsivity and inattention are based on the validated questionnaire by Vas et al.^60^ We earlier reduced the nine unwanted behavior traits into four larger factors, which were utilized in the current study: fear-related behavior, aggression, fear-aggression, and impulsivity/inattention.^46^ The personality and behavior questionnaire has shown good inter-rater and test-retest reliability, convergent and discriminant validity, and adequate internal consistency of all factors in a previous study by Salonen et al.^35^ For a detailed description of the questionnaire, see the Supplementary Material of Salonen et al.^35^ Factor scores that are used in this study were calculated for each dog based on the item loadings from the factor analysis by Salonen et al.^35^

#### Cat personality and behavior

To collect behavioral, personality and background information from cats, we used the validated questionnaire of feline behavior and personality.^34^ The behavior section includes 138 statements that ask individuals to indicate their level of agreement with each item. Based on a dataset of over 4 300 responses, Mikkola et al.^34^ previously utilized factor analysis, reduced items into 84 statements based on reliability, missingness and item loadings, and discovered a structure of five personality factors (fearfulness, activity/playfulness, aggression toward humans, sociability toward humans, sociability toward cats), and two unwanted behavior factors (excessive grooming, and litterbox issues). See Table S14 for sample items. The questionnaire has shown good levels of inter-rater and test-retest reliability, convergent validity, and discriminant validity in a previous study by Mikkola et al.^34^ For a detailed description of the questionnaire, see the Supplementary Material of Mikkola et al.^34^ Factor scores that are used in this study were calculated for each cat based on the item loadings from the factor analysis by Mikkola et al.^34^

### Quantification and statistical analysis

We used structural equation modeling (SEM) with the package *lavaan*^61^ to examine the association of owner personality and well-being and pet personality and unwanted behavior with attachment insecurity. We also included three covariates related to the owner: gender, age group (as a numerical value), and children in the household (yes/no) (Table S1). We fit models for dog owners and cat owners separately. We defined eight competing structures which differed by type of attachment scores (original scales [which we scaled as they showed much larger variances than other variables]/latent variables), the number of owner personality traits included (based on previous literature,^2^^,^^31^^,^^32^^,^^33^ and type of latent well-being variable (single latent variable/two latent variables; Figure S1). We selected the final structures for dog and cat owners by comparing CFI, TLI, RMSEA, and SRMR of these competing structures. All competing models included one or two latent well-being variables and regressions from owner personality, owner wellbeing, pet behavior, and covariates to attachment anxiety and insecurity. All models also included regressions from age and gender to owner personality traits and regressions from owner personality traits to latent well-being variable(s). Models for dog owners also included the four latent dog unwanted behavior variables and regressions from these latent variables to attachment scales. All models also included covariance between some owner personality traits, between some pet personality traits, between dog unwanted behavior traits, between age and well-being, and between gender and well-being (Tables S7 and S8). In dogs, personality trait insecurity was highly associated with fear-related behavior, personality trait aggressiveness/dominance with aggression, and personality trait training focus with impulsivity/inattention, leading to extremely wide confidence limits. Therefore, we compared models including only unwanted behavioral traits and in the final model, only included personality traits not highly linked to unwanted behaviors but associated with attachment styles: human sociability in attachment avoidance and perseverance in attachment anxiety. We used robust maximum likelihood estimator and handled missing data with full information maximum likelihood with the option that does not delete cases that have missing data in exogenous variables. Finally, we extracted the RMSEA of the null models for dog owners and cat owners with the package semTools^62^^,^^63^ We set the significance level at p value <0.05. All statistical analyses were conducted in R version 4.2.1.^64^ The data assumptions were checked to ensure compliance with the statistical approach. Assumptions of SEM include adequate sample size (minimum of 200 observations^65^) and correctly specified. The sample size requirement was met and we assessed the specification of the model by model fit indices (CFI, TLI, RMSEA, and SRMR).

## Supplemental information

Document S1. Figure S1 and Tables S1–S14

Data S1. R-code

## Footnotes

Supplemental information can be found online at https://doi.org/10.1016/j.isci.2023.108423.

## References

[1] Zilcha-Mano S., Mikulincer M., Shaver P.R. (2012). Pets as safe havens and secure bases: The moderating role of pet attachment orientations. J. Res. Pers..

[2] Zilcha-Mano S., Mikulincer M., Shaver P.R. (2011). An attachment perspective on human-pet relationships: Conceptualization and assessment of pet attachment orientations. J. Res. Pers..

[3] Cavanaugh L.A., Leonard H.A., Scammon D.L. (2008). A tail of two personalities: How canine companions shape relationships and well-being. J. Bus. Res..

[4] Coy A.E., Green J.D., Behler A.M.C. (2021). Why Can’t I Resist Those “Puppy Dog” (or “Kitty Cat”) Eyes? A Study of Owner Attachment and Factors Associated with Pet Obesity. Animals.

[5] Coy A.E., Green J.D. (2018). Treating Pets Well: The Role of Attachment Anxiety and Avoidance. Hum. Anim. Interact Bull..

[6] Teo J.T., Thomas S.J. (2019). Psychological Mechanisms Predicting Wellbeing in Pet Owners: Rogers’ Core Conditions versus Bowlby’s Attachment. Anthrozoös.

[7] Beck L., Madresh E.A. (2008). Romantic partners and four-legged friends: An extension of attachment theory to relationships with pets. Anthrozoös.

[8] Stammbach K.B., Turner D.C. (1999). Understanding the human-cat relationship: Human social support or attachment. Anthrozoös.

[9] Archer J., Ireland J.L. (2011). The development and factor structure of a questionnaire measure of the strength of attachment to pet dogs. Anthrozoös.

[10] Solomon J., Beetz A., Schöberl I., Gee N., Kotrschal K. (2019). Attachment security in companion dogs: adaptation of Ainsworth’s strange situation and classification procedures to dogs and their human caregivers. Attach. Hum. Dev..

[11] Julius H., Beetz A., Kortschal D.T., Uvnäs-Modberg K. (2012).

[12] Bartholomew K., Horowitz L.M. (1991). Attachment styles among young adults: A test of a four-category model. J. Pers. Soc. Psychol..

[13] Gobbo E., Zupan M. (2020). Dogs’ sociability, owners’ neuroticism and attachment style to pets as predictors of dog aggression. Animals.

[14] Powell L., Stefanovski D., Siracusa C., Serpell J. (2021). Owner Personality, Owner-Dog Attachment, and Canine Demographics Influence Treatment Outcomes in Canine Behavioral Medicine Cases. Front. Vet. Sci..

[15] Van Herwijnen I.R., Van Der Borg J.A.M., Naguib M., Beerda B. (2018). Dog ownership satisfaction determinants in the owner-dog relationship and the dog’s behaviour. PLoS One.

[16] Konok V., Kosztolányi A., Rainer W., Mutschler B., Halsband U., Miklósi Á. (2015). Influence of owners’ attachment style and personality on their dogs’ (Canis familiaris) separation-related disorder. PLoS One.

[17] Meyer I., Forkman B. (2014). Dog and owner characteristics affecting the dog-owner relationship. J. Vet. Behav. Clin. Appl. Res..

[18] Kwan J.Y., Bain M.J. (2013). Owner Attachment and Problem Behaviors Related to Relinquishment and Training Techniques of Dogs. J. Appl. Anim. Welfare Sci..

[19] Walker S.L., Weng H.-Y., Ogata N., Anderson K.G. (2022). Do Canine and Human Personality Assessments Predict Successful Adoptions?. Anthrozoös.

[20] Chopik W.J., Weaver J.R. (2019). Old dog, new tricks: Age differences in dog personality traits, associations with human personality traits, and links to important outcomes. J. Res. Pers..

[21] McConnell A.R., Brown C.M., Shoda T.M., Stayton L.E., Martin C.E. (2011). Friends with benefits: On the positive consequences of pet ownership. J. Pers. Soc. Psychol..

[22] Elvers G.C., Lawriw A.N. (2019). The Behavioral Style of the Cat Predicts Owner Satisfaction. Anthrozoös.

[23] Bennett P.C., Rutter N.J., Woodhead J.K., Howell T.J. (2017). Assessment of domestic cat personality, as perceived by 416 owners, suggests six dimensions. Behav. Process..

[24] Evans R., Lyons M., Brewer G., Bethell E. (2021). A domestic cat (Felis silvestris catus) model of triarchic psychopathy factors: Development and initial validation of the CAT-Tri+ questionnaire. J. Res. Pers..

[25] Evans R., Lyons M., Brewer G., Tucci S. (2019). The purrfect match: The influence of personality on owner satisfaction with their domestic cat (Felis silvestris catus). Pers. Individ. Dif..

[26] Tackett J.L., Lahey B.B., Widiger T.A. (2016). The Oxford Handbook of the Five Factor Model.

[27] Wilt J., Revelle W., Widiger T.A. (2016). The Oxford Handbook of the Five Factor Model.

[28] Sutin A.R., Widiger T.A. (2015). The Oxford Handbook of the Five Factor Model.

[29] Graziano W.G., Tobin R.M., Widiger T.A. (2016). The Oxford Handbook of the Five Factor Model.

[30] Jackson J.J., Roberts B.W., Widiger T.A. (2015). The Oxford Handbook of the Five Factor Model.

[31] Noftle E.E., Shaver P.R. (2006). Attachment dimensions and the big five personality traits: Associations and comparative ability to predict relationship quality. J. Res. Pers..

[32] Reevy G.M., Delgado M.M. (2015). Are Emotionally Attached Companion Animal Caregivers Conscientious and Neurotic? Factors That Affect the Human–Companion Animal Relationship. J. Appl. Anim. Welfare Sci..

[33] Reevy G.M., Delgado M.M. (2020). The Relationship Between Neuroticism Facets, Conscientiousness, and Human Attachment to Pet Cats. Anthrozoös.

[34] Mikkola S., Salonen M., Hakanen E., Sulkama S., Lohi H. (2021). Reliability and Validity of Seven Feline Behavior and Personality Traits. Animals.

[35] Salonen M., Mikkola S., Hakanen E., Sulkama S., Puurunen J., Lohi H. (2021). Reliability and validity of a dog personality and unwanted behavior survey. Animals.

[36] Diener E., Emmons R.A., Larsen R.J., Griffin S. (1985). The Satisfaction With Life Scale. J. Pers. Assess..

[37] Cohen S., Kamarck T., Mermelstein R. (1983). A Global Measure of Perceived Stress. J. Health Soc. Behav..

[38] Stewart-Brown S., Tennant A., Tennant R., Platt S., Parkinson J., Weich S. (2009). Internal construct validity of the Warwick-Edinburgh Mental Well-Being Scale (WEMWBS): A Rasch analysis using data from the Scottish Health Education Population Survey. Health Qual Life Outcomes.

[39] Spitzer R.L., Kroenke K., Williams J.B.W., Löwe B. (2006). A Brief Measure for Assessing Generalized Anxiety Disorder. Arch. Intern. Med..

[40] Kohout F.J., Berkman L.F., Evans D.A., Cornoni-Huntley J. (1993). Two Shorter Forms of the CES-D Depression Symptoms Index. J. Aging Health.

[41] Kenny D. (2020). https://davidakenny.net/cm/fit.htm.

[42] McCrae R.R., Costa P.T. (1987). Validation of the five-factor model of personality across instruments and observers. J. Pers. Soc. Psychol..

[43] Roberts B.W., Kuncel N.R., Shiner R., Caspi A., Goldberg L.R. (2007). The Power of Personality: The Comparative Validity of Personality Traits, Socioeconomic Status, and Cognitive Ability for Predicting Important Life Outcomes. Perspect. Psychol. Sci..

[44] Wright J.C., Smith A., Daniel K., Adkins K. (2007). Dog breed stereotype and exposure to negative behavior: Effects on perceptions of adoptability. J. Appl. Anim. Welf. Sci..

[45] Sulkama S., Puurunen J., Salonen M., Mikkola S., Hakanen E., Araujo C., Lohi H. (2021). Canine hyperactivity, impulsivity, and inattention share similar demographic risk factors and behavioural comorbidities with human ADHD. Transl. Psychiatry.

[46] Salonen M., Mikkola S., Hakanen E., Sulkama S., Puurunen J., Lohi H. (2022). Personality traits associate with behavioral problems in pet dogs. Transl. Psychiatry.

[47] Matteson L.K., McGue M., Iacono W.G. (2013). Shared Environmental Influences on Personality: A Combined Twin and Adoption Approach. Behav. Genet..

[48] Roberts B.W., DelVecchio W.F. (2000). The rank-order consistency of personality traits from childhood to old age: A quantitative review of longitudinal studies. Psychol. Bull..

[49] Fraley R.C., Roisman G.I. (2019). The development of adult attachment styles: four lessons. Curr. Opin. Psychol..

[50] Curb L.A., Abramson C.I., Grice J.W., Kennison S.M. (2013). The relationship between personality match and pet satisfaction among dog owners. Anthrozoös.

[51] Zeigler-Hill V., Highfill L. (2010). Applying the interpersonal circumplex to the behavioral styles of dogs and cats. Appl. Anim. Behav. Sci..

[52] Dotson M.J., Hyatt E.M. (2008). Understanding dog-human companionship. J. Bus. Res..

[53] Smolkovic I., Fajfar M., Mlinaric V. (2012). Attachment to Pets and Interpersonal Relationships: Can a four-legged friend replace a two-legged one?. J. Eur. Psychol. Stud..

[54] Winefield H.R., Black A., Chur-Hansen A. (2008). Health effects of ownership of and attachment to companion animals in an older population. Int. J. Behav. Med..

[55] Woodward L.E., Bauer A.L. (2007). People and their pets: A relational perspective on interpersonal complementarity and attachment in companion animal owners. Soc. Anim..

[56] Kotrschal K., Schöberl I., Bauer B., Thibeaut A.M., Wedl M. (2009). Dyadic relationships and operational performance of male and female owners and their male dogs. Behav. Process..

[57] Konstabel K., Lönnqvist J.E., Walkowitz G., Konstabel K., Verkasalo M. (2012). The “Short Five” (S5): Measuring personality traits using comprehensive single items. Eur. J. Pers..

[58] Lönnqvist J., Verkasalo M., Leikas S. (2008). Viiden suuren persoonallisuusfaktorin 10, 60, ja 300 osion julkiset mittarit. Psykologia.

[59] Dai S., Wang X., Svetina D. (2021). https://CRAN.R-project.org/package=TestDataImputation.

[60] Vas J., Topál J., Péch É., Miklósi Á. (2007). Measuring attention deficit and activity in dogs: A new application and validation of a human ADHD questionnaire. Appl. Anim. Behav. Sci..

[61] Rosseel Y. (2012). lavaan: An R Package for Structural Equation Modeling. J Stat Softw.

[62] Jorgensen T., Pornprasertmanit S., Schoemann A. (2022). https://CRAN.R-project.org/package=semTools.

[63] Kenny D.A., Kaniskan B., McCoach D.B. (2015). The Performance of RMSEA in Models With Small Degrees of Freedom. Sociol Methods Res.

[64] R Core Team (2022).

[65] Kline R.B. (2011).

