# Supplementary material for: Pet and owner personality and mental wellbeing associate with attachment to cats and dogs
Source: iScience. 2023 Nov 9;26(12):108423. doi: 10.1016/j.isci.2023.108423 (PMC10709106; doi:10.1016/j.isci.2023.108423)
Supplement: Document S1. Figure S1 and Tables S1–S14 [file mmc1.pdf]

iScience, Volume *26*

Supplemental information

Pet and owner personality and mental wellbeing associate with attachment to cats and dogs

Aada Ståhl, Milla Salonen, Emma Hakanen, Salla Mikkola, Sini Sulkama, Jari Lahti, and Hannes Lohi

Supplementary Table S1. Descriptive statistics of categorical variables, Related to STAR Methods.

Variable	Level	Dogs N	%	Cats N	%
Gender of the owner	Women	2359	92.69	719	91.24
	Men/other/did not say	186	7.31	69	8.76
Age of the owner	1 = under 25	143	5.62	48	6.09
	2 = 25-29 years	349	13.71	130	16.50
	3 = 30-34 years	332	13.05	113	14.34
	4 = 35-39 years	309	12.14	92	11.68
	5 = 40-44 years	322	12.65	91	11.55
	6 = 45-49 years	261	10.26	90	11.42
	7 = 50-54 years	272	10.69	70	8.88
	8 = 55-59 years	219	8.61	50	6.35
	9 = over 60	240	9.43	63	8.00
	Unknown/ did not say	98	3.85	41	5.23
Parental status of the owner	No children	1861	73.12	589	74.75
	Children	565	22.20	92	11.68
	Missing	119	4.68	107	13.58

Supplementary Table S2. Descriptive statistics of continuous variables, species = dogs, Related to STAR Methods.

Category	Variable	Range	Mean	SD	% Missing
Attachment insecurity	Avoidance trait	13 - 69	19.49	7.00	12.81
	Anxiety trait	13 - 77	30.00	10.56	12.81
Attachment insecurity items	Avoid1 (originally: 1[1])	1 - 7	1.29	0.87	12.85
	Anx1 (originally: 2[1])	1 - 7	5.27	1.71	12.81
	Avoid2 (originally: 3[1])	1 - 7	1.41	0.93	12.81
	Anx2 (originally: 4[1])	1 - 7	1.94	1.42	12.81
	Avoid3 (originally: 5[1])	1 - 7	1.61	1.13	12.85
	Anx3 (originally: 6[1])	1 - 7	1.44	0.93	12.85
	Avoid4 (originally: 7[1])	1 - 7	1.94	1.30	12.85
	Anx4 (originally: 8[1])	1 - 7	4.34	1.90	12.85
	Avoid5 (originally: 9[1])	1 - 7	1.34	0.85	12.81
	Anx5 (originally: 10[1])	1 - 7	1.67	1.28	12.81
	Avoid6 (originally: 11[1])	1 - 7	1.15	0.59	12.85
	Anx6 (originally: 12[1])	1 - 6	1.18	0.60	12.81
	Avoid7 (originally: 13[1])	1 - 7	1.23	0.74	12.81
	Anx7 (originally: 14[1])	1 - 7	1.44	0.95	12.85
	Avoid8 (originally: 15[1])	1 - 7	2.49	1.60	12.81
	Anx8 (originally: 16[1])	1 - 7	2.49	1.77	12.85
	Avoid9 (originally: 17[1])	1 - 7	1.29	0.75	12.81
	Anx9 (originally: 18[1])	1 - 7	1.81	1.33	12.93
	Avoid10 (originally: 19[1])	1 - 6	1.32	0.77	12.81

	Anx10 (originally: 20[1])	1 - 7	1.84	1.37	12.85
	Avoid11 (originally: 21[1])	1 - 7	1.30	0.87	12.85
	Anx11 (originally: 22[1])	1 - 7	2.87	1.98	12.89
	Avoid12 (originally: 23[1])	1 - 7	1.26	0.78	12.81
	Anx12 (originally: 24[1])	1 - 7	2.07	1.55	12.85
	Avoid13 (originally: 25[1])	1 - 7	1.87	1.23	12.81
	Anx13 (originally: 26[1])	1 - 7	1.63	1.16	12.81
Dog personality trait	Insecurity score	-1.58 - 3.49	0.04	1.00	1.81
	Training focus score	-4.21 - 2.48	0.02	1.00	1.81
	Energy score	-3.88 - 2.08	0.04	1.00	1.81
	Aggressiveness/ dominance score	-1.56 - 3.78	-0.01	1.00	1.81
	Human sociability score	-4.91 - 1.65	0.03	0.97	1.81
	Dog sociability score	-3.08 - 1.96	0.03	1.01	1.81
	Perseverance score	-4.16 - 3.69	-0.02	1.00	1.81
Dog unwanted behaviour trait	Noise sensitivity score	-1.03 - 7.36	0.03	1.18	15.60
	Fearfulness score	-1.51 - 4.50	0.07	1.08	8.57
	Barking score	-1.36 - 4.61	0.00	1.00	8.57
	Stranger directed aggression score	-1.11 - 7.67	0.00	0.95	8.57
	Owner directed aggression score	-0.93 - 8.82	0.00	0.96	8.57
	Dog directed aggression score	-1.99 - 4.18	-0.02	0.99	8.57
	Fear of surfaces/ heights score	-0.69 - 6.20	0.03	1.09	18.62
	Separation-related behaviour score	-0.63 - 12.81	-0.02	1.07	14.77
	Inattention score	-1.74 - 4.33	0.00	0.96	9.98
	Hyperactivity/ impulsivity score	-1.65 - 4.75	0.02	0.99	9.98
Owner personality trait	Agreeableness score	-2.58 - 2.83	1.25	0.70	3.81
	Conscientiousness score	-1.83 - 2.92	1.13	0.82	3.81
	Extraversion score	-2.75 - 2.75	0.14	0.96	3.81
	Neuroticism score	-2.92 - 2.83	-0.65	1.11	3.81
	Openness score	-1.83 - 3.00	1.01	0.85	3.81
Owner well-being trait	Life satisfaction score	5 - 35	24.63	5.93	29.27
	Perceived stress score	0 - 39	12.50	7.20	29.27
	Wellbeing score	8 - 35	26.53	4.24	29.27
	Depressive symptoms score	0 - 30	6.92	5.64	29.27
	Anxiety symptoms score	0 - 21	4.33	4.16	29.27

[1] Zilcha-Mano, S., Mikulincer, M., & Shaver, P. R. (2011). An attachment perspective on human–pet relationships: Conceptualization and assessment of pet attachment orientations. *Journal of Research in Personality*, *45*(4), 345-357.

Supplementary Table S3. Descriptive statistics of continuous variables, species = cats, Related to STAR Methods.

Category	Variable	Range	Mean	SD	% Missing
Attachment insecurity	Avoidance	13 - 60	17.97	5.09	12.69
	Anxiety	13 - 79	31.98	11.29	12.69
Attachment insecurity items	Avoid1	1 - 7	1.22	0.87	12.69
	Anx1	1 - 7	5.33	1.76	12.82
	Avoid2	1 - 7	1.21	0.71	12.69
	Anx2	1 - 7	2.21	1.54	12.69
	Avoid3	1 - 7	1.39	0.86	12.69
	Anx3	1 - 6	1.26	0.69	12.69
	Avoid4	1 - 7	1.86	1.15	12.69
	Anx4	1 - 7	4.62	1.89	12.69
	Avoid5	1 - 5	1.40	0.86	12.69
	Anx5	1 - 7	2.15	1.61	12.69
	Avoid6	1 - 7	1.12	0.54	12.82
	Anx6	1 - 7	1.19	0.66	12.69
	Avoid7	1 - 6	1.16	0.55	12.69
	Anx7	1 - 7	1.62	1.10	12.69
	Avoid8	1 - 7	2.19	1.43	12.69
	Anx8	1 - 7	2.71	1.85	12.69
	Avoid9	1 - 7	1.21	0.66	12.82
	Anx9	1 - 7	1.69	1.18	12.69
	Avoid10	1 - 7	1.19	0.60	12.69
	Anx10	1 - 7	2.00	1.48	12.69
	Avoid11	1 - 6	1.17	0.58	12.69
	Anx11	1 - 7	3.05	2.04	12.69
	Avoid12	1 - 5	1.12	0.45	12.69
	Anx12	1 - 7	2.37	1.73	12.69
	Avoid13	1 - 6	1.71	1.03	12.69
	Anx13	1 - 7	1.80	1.28	12.69
Cat personality trait	Fearfulness	-1.68 - 3.89	0.12	0.98	0.00
	Activity/playfulness	-3.67 - 2.51	0.00	0.97	0.00
	Human aggression	-1.09 - 4.73	-0.08	0.86	0.00
	Human sociability	-3.82 - 2.73	0.01	0.98	0.00
	Sociability toward cats	-4.46 - 2.10	0.02	0.94	0.00
Cat unwanted behaviour trait	Excessive grooming	-1.23 - 6.95	0.00	0.99	0.00
	Litterbox issues	-1.33 - 4.33	-0.05	0.93	0.00
Human personality trait	Agreeableness score	-1.63 - 2.83	1.22	0.72	5.20
	Conscientiousness score	-2.00 - 2.92	1.02	0.88	5.20
	Extraversion score	-2.42 - 2.50	-0.08	0.95	5.20
	Neuroticism score	-2.83 - 2.25	-0.32	1.12	5.20
	Openness score	-1.50 - 2.92	1.18	0.84	5.20

Owner well-being trait	Life satisfaction score	5 - 35	23.23	6.53	33.00
	Perceived stress score	1 - 33	13.63	7.18	33.00
	Wellbeing score	12 - 35	25.71	4.33	33.00
	Depressive symptoms score	0 - 30	7.93	6.00	33.00
	Anxiety symptoms score	0 - 21	5.01	4.43	33.12

Supplementary Table S4. Model fit of competing SEM models. CFI = comparative fit index, TLI = Tucker-Lewis index, RMSEA = root mean square error of approximation, SRMR = standardized root mean square residual, Related to STAR Methods.

	**Dogs**				**Cats**			
**Competing model**	**CFI**	**TLI**	**RMSEA**	**SRMR**	**CFI**	**TLI**	**RMSEA**	**SRMR**
Model 1	0.907	0.884	0.050	0.065	0.881	0.842	0.060	0.060
Model 2	0.763	0.744	0.055	0.071	0.669	0.640	0.068	0.082
Model 3	0.903	0.882	0.051	0.067	0.883	0.848	0.061	0.060
Model 4	0.761	0.744	0.055	0.071	0.669	0.642	0.069	0.082
Model 5	0.866	0.829	0.061	0.078	0.819	0.752	0.076	0.076
Model 6	0.743	0.722	0.057	0.074	0.644	0.611	0.071	0.085
Model 7	0.862	0.829	0.061	0.079	0.820	0.758	0.077	0.077
Model 8	0.741	0.722	0.057	0.075	0.644	0.613	0.072	0.086

Model 1: one latent well-being trait, attachment anxiety and avoidance as scales, more personality traits
Model 2: one latent well-being trait, attachment anxiety and avoidance as latent traits, more personality traits
Model 3: one latent well-being trait, attachment anxiety and avoidance as scales, less personality traits
Model 4: one latent well-being trait, attachment anxiety and avoidance as latent traits, less personality traits
Model 5: two latent well-being traits, attachment anxiety and avoidance as scales, more personality traits
Model 6: two latent well-being traits, attachment anxiety and avoidance as latent traits, more personality traits
Model 7: two latent well-being traits, attachment anxiety and avoidance as scales, less personality traits
Model 8: two latent well-being traits, attachment anxiety and avoidance as latent traits, less personality traits

Supplementary Table S5. Unstandardized estimates, standardized estimates, Z-scores, P-values, and 95% confidence limits (CL, for standardized estimates) for the regressions of the SEM model for dog owners, Related to STAR Methods. All P-values are corrected for false discovery rate (FDR). Significant (<0.05) P-values are in bold. CL = confidence limit.

**Outcome variable**	**Explanatory variable**	**Unstand-ardized estimate**	**Stand-ardized estimate**	**Z-score**	**P-value**	**Lower 95% CL**	**Upper 95 % CL**
Attachment avoidance	Agreeableness	-0.120	-0.085	-3.43	**0.002**	-0.134	-0.036
	Conscientiousness	-0.072	-0.059	-2.05	0.060	-0.116	-0.002
	Extraversion	-0.065	-0.062	-2.39	**0.026**	-0.113	-0.011
	Neuroticism	-0.078	-0.085	-2.18	**0.045**	-0.162	-0.008
	Openness	-0.016	-0.014	-0.56	0.661	-0.062	0.034
	Fear-aggression	-0.074	-0.037	-1.14	0.320	-0.101	0.027
	Aggression	0.356	0.158	2.74	**0.010**	0.045	0.272
	Impulsivity/inattention	0.341	0.203	6.48	**<0.001**	0.141	0.264
	Owner's gender	-0.188	-0.050	-2.04	0.060	-0.097	-0.002
	Owner's age group	-0.017	-0.043	-1.87	0.087	-0.087	0.002
	Children in the household	0.244	0.104	4.79	**<0.001**	0.062	0.147
	Total wellbeing	-0.030	-0.122	-2.97	**0.005**	-0.202	-0.041
	Dog's human sociability	-0.113	-0.112	-4.84	**<0.001**	-0.157	-0.066
Attachment anxiety	Agreeableness	-0.027	-0.019	-0.92	0.434	-0.061	0.022
	Conscientiousness	-0.014	-0.011	-0.50	0.698	-0.055	0.033
	Extraversion	-0.015	-0.014	-0.67	0.587	-0.057	0.028
	Neuroticism	0.228	0.251	6.63	**<0.001**	0.176	0.325
	Fear-aggression	-0.038	-0.019	-0.89	0.452	-0.061	0.023
	Fear-related behavior	0.103	0.068	2.39	**0.026**	0.012	0.124
	Owner's gender	0.005	0.001	0.07	0.951	-0.037	0.040
	Owner's age group	-0.009	-0.021	-1.02	0.386	-0.063	0.020
	Children in the household	-0.290	-0.124	-6.45	**<0.001**	-0.162	-0.086
	Total wellbeing	-0.045	-0.182	-4.90	**<0.001**	-0.255	-0.109
	Dog's perseverance	0.042	0.042	2.11	0.052	0.003	0.082
Total wellbeing	Neuroticism	-2.714	-0.734	-48.14	**<0.001**	-0.764	-0.704
	Extraversion	-0.002	-0.001	-0.03	0.979	-0.040	0.039
Neuroticism	Owner's gender	0.215	0.052	2.49	**0.021**	0.011	0.092
	Owner's age group	-0.154	-0.343	-21.30	**<0.001**	-0.375	-0.311
Agreeableness	Owner's gender	0.301	0.111	4.50	**<0.001**	0.063	0.160
	Owner's age group	0.036	0.124	6.46	**<0.001**	0.087	0.162
Conscientiousness	Owner's age group	0.048	0.141	7.24	**<0.001**	0.103	0.180

Supplementary Table S6. Unstandardized estimates, standardized estimates, Z-scores, P-values, and 95% confidence limits (CL, for standardized estimates) for the regressions of the SEM model for cat owners, Related to STAR Methods. All P-values are corrected for false discovery rate (FDR). Significant (<0.05) P-values are in bold. CL = confidence limit.

**Outcome variable**	**Explanatory variable**	**Unstand-ardized estimate**	**Stand-ardized estimate**	**Z-score**	**P-value**	**Lower 95% CL**	**Upper 95 % CL**
Attachment avoidance	Agreeableness	-0.098	-0.071	-1.89	0.084	-0.144	0.003
	Conscientiousness	-0.087	-0.077	-1.83	0.093	-0.159	0.005
	Extraversion	-0.018	-0.017	-0.41	0.754	-0.096	0.063
	Neuroticism	-0.086	-0.095	-1.43	0.198	-0.226	0.035
	Openness	0.011	0.009	0.23	0.878	-0.069	0.087
	Cat's fearfulness	0.013	0.013	0.32	0.823	-0.065	0.091
	Cat's human aggression	0.044	0.038	1.01	0.387	-0.035	0.110
	Cat's activity/playfulness	-0.061	-0.059	-1.40	0.208	-0.141	0.023
	Cat's cat sociability	-0.003	-0.003	-0.09	0.945	-0.072	0.065
	Cat's human sociability	-0.098	-0.096	-2.54	**0.018**	-0.170	-0.022
	Cat's litterbox issues	0.041	0.038	0.83	0.485	-0.052	0.129
	Cat's excessive grooming	0.034	0.034	0.64	0.609	-0.071	0.139
	Owner's gender	-0.043	-0.012	-0.31	0.832	-0.090	0.065
	Owner's age group	0.005	0.013	0.29	0.839	-0.073	0.099
	Children in the household	0.228	0.078	1.66	0.131	-0.014	0.171
	Total wellbeing	-0.026	-0.122	-1.74	0.111	-0.259	0.015
Attachment anxiety	Agreeableness	-0.021	-0.015	-0.47	0.716	-0.080	0.049
	Conscientiousness	0.102	0.091	2.28	**0.036**	0.013	0.168
	Extraversion	0.074	0.069	1.92	0.079	-0.001	0.140
	Neuroticism	0.343	0.380	6.34	**<0.001**	0.263	0.498
	Cat's fearfulness	0.065	0.064	1.93	0.078	-0.001	0.129
	Cat's human aggression	0.063	0.054	1.72	0.116	-0.008	0.116
	Cat's activity/playfulness	0.090	0.088	2.63	**0.015**	0.022	0.153
	Cat's cat sociability	-0.060	-0.057	-1.50	0.176	-0.131	0.018
	Cat's human sociability	-0.009	-0.008	-0.26	0.859	-0.071	0.054
	Cat's litterbox issues	0.057	0.053	1.64	0.136	-0.010	0.116
	Cat's excessive grooming	-0.005	-0.005	-0.16	0.913	-0.070	0.060
	Owner's gender	-0.117	-0.033	-0.97	0.411	-0.101	0.034
	Owner's age group	0.001	0.001	0.04	0.973	-0.071	0.074
	Children in the household	-0.312	-0.108	-3.24	**0.002**	-0.173	-0.043
	Total wellbeing	-0.062	-0.287	-4.69	**<0.001**	-0.408	-0.167
Total wellbeing	Neuroticism	-3.087	-0.738	-33.40	**<0.001**	-0.781	-0.694
	Extraversion	0.023	0.005	0.15	0.917	-0.059	0.068
Neuroticism	Owner's gender	0.326	0.084	1.95	0.076	-0.001	0.168
	Owner's age group	-0.193	-0.417	-15.19	**<0.001**	-0.470	-0.363
Agreeableness	Owner's gender	0.234	0.092	1.87	0.087	-0.005	0.190
	Owner's age group	0.005	0.018	0.49	0.707	-0.054	0.089

Conscientiousness	Owner's age group	0.097	0.262	7.84	**<0.001**	0.197	0.328

Supplementary Table S7. Unstandardized estimates, standardized estimates, Z-scores, P-values, and 95% confidence limits (CL, for standardized estimates) for the covariances of the SEM model for dog owners, Related to STAR Methods. All P-values are corrected for false discovery rate (FDR). Significant (<0.05) P-values are in bold. CL = confidence limit.

**Variable 1**	**Variable 2**	**Unstand-ardized estimate**	**Stand-ardized estimate**	**Z-score**	**P-value**	**Lower 95% CL**	**Upper 95 % CL**
Total well-being	Owner's gender	0.018	0.025	0.90	0.445	-0.029	0.080
Fear-aggression	Aggression	0.113	0.511	13.03	**<0.001**	0.434	0.588
	Fear-related behaviour	0.072	0.221	5.73	**<0.001**	0.146	0.297
	Impulsivity/inattention	0.056	0.19	8.82	**<0.001**	0.148	0.232
Fear-related behaviour	Impulsivity/inattention	0.112	0.293	5.59	**<0.001**	0.190	0.396
	Aggression	0.092	0.318	5.82	**<0.001**	0.211	0.425
Aggression	Impulsivity/inattention	0.080	0.308	8.85	**<0.001**	0.240	0.377
Neuroticism	Conscientiousness	-0.399	-0.484	-29.63	**<0.001**	-0.516	-0.452
	Extraversion	-0.414	-0.427	-25.18	**<0.001**	-0.460	-0.393
	Agreeableness	-0.048	-0.068	-3.48	**0.002**	-0.107	-0.030
Openness	Extraversion	0.259	0.325	19.61	**<0.001**	0.292	0.357
	Agreeableness	0.156	0.269	13.90	**<0.001**	0.231	0.307
Conscientiousness	Extraversion	0.155	0.201	10.75	**<0.001**	0.165	0.238

Supplementary Table S8. Unstandardized estimates, standardized estimates, Z-scores, P-values, and 95% confidence limits (CL, for standardized estimates) for the covariances of the SEM model for cat owners, Related to STAR Methods. All P-values are corrected for false discovery rate (FDR). Significant (<0.05) P-values are in bold. CL = confidence limit.

**Variable 1**	**Variable 2**	**Unstand-ardized estimate**	**Stand-ardized estimate**	**Z-score**	**P-value**	**Lower 95% CL**	**Upper 95 % CL**
Total wellbeing	Owner's gender	0.044	0.050	0.75	0.532	-0.081	0.181
Neuroticism	Conscientiousness	-0.382	-0.451	-14.22	**<0.001**	-0.514	-0.389
	Extraversion	-0.352	-0.377	-11.88	**<0.001**	-0.440	-0.315
	Agreeableness	-0.057	-0.080	-2.26	**0.037**	-0.150	-0.011
Extraversion	Openness	0.253	0.324	10.29	**<0.001**	0.263	0.386
Conscientiousness	Extraversion	0.118	0.148	4.35	**<0.001**	0.081	0.215
Agreeableness	Openness	0.151	0.254	6.65	**<0.001**	0.179	0.329
Cat's activity/ playfulness	Cat's cat sociability	0.255	0.282	8.52	**<0.001**	0.217	0.347
Cat's human aggression	Cat's cat sociability	-0.182	-0.227	-5.15	**<0.001**	-0.313	-0.140
Cat's cat sociability	Cat's litterbox issues	-0.070	-0.081	-1.91	0.081	-0.164	0.002
Cat's activity playfulness	Cat's human sociability	0.198	0.210	6.30	**<0.001**	0.145	0.276
Cat's fearfulness	Cat's human sociability	-0.168	-0.176	-5.26	**<0.001**	-0.242	-0.110
	Cat's human aggression	0.209	0.250	7.20	**<0.001**	0.182	0.318
Cat's litterbox issues	Cat's excessive grooming	0.176	0.193	6.03	**<0.001**	0.130	0.256

Supplementary Table S9. Unstandardized estimates, standardized estimates, Z-scores, P-values, and 95% confidence limits (CL, for standardized estimates) for the latent variables of the SEM model for dog owners, Related to STAR Methods. All P-values are corrected for false discovery rate (FDR). Significant (<0.05) P-values are in bold. CL = confidence limit.

Latent variable	Observed variable	Unstand-ardized estimate	Stand-ardized estimate	Z-score	P-value	Lower 95% CL	Upper 95 % CL
Total wellbeing	Life satisfaction	1.000	0.682	43.45	**<0.001**	0.651	0.713
	Perceived stress	-1.611	-0.908	-148.49	**<0.001**	-0.920	-0.896
	Wellbeing	0.885	0.847	96.92	**<0.001**	0.830	0.864
	CESD-10	-1.247	-0.898	-140.40	**<0.001**	-0.910	-0.885
	GAD-7	-0.844	-0.823	-80.92	**<0.001**	-0.843	-0.803
Fear-aggression	Dog's fearfulness	1.000	0.464	16.82	**<0.001**	0.410	0.519
	Dog's barking	1.991	0.993	30.56	**<0.001**	0.929	1.057
	Dog's stranger aggression	0.669	0.353	8.97	**<0.001**	0.276	0.430
Fear-related behaviour	Dog's noise sensitivity	1.000	0.557	15.29	**<0.001**	0.485	0.628
	Dog's separation behavior	0.511	0.312	5.87	**<0.001**	0.208	0.416
	Dog's surface phobia	0.714	0.428	10.21	**<0.001**	0.346	0.510
	Dog's fearfulness	0.648	0.393	10.63	**<0.001**	0.321	0.466
Aggression	Dog's owner aggression	1.000	0.456	10.00	**<0.001**	0.367	0.546
	Dog's dog aggression	1.288	0.574	16.16	**<0.001**	0.505	0.644
	Dog's stranger aggression	1.003	0.465	9.17	**<0.001**	0.365	0.564
Impulsivity/ inattention	Dog's inattention	1.000	0.611	17.65	**<0.001**	0.543	0.679
	Dog's impulsivity	1.597	0.947	20.76	**<0.001**	0.858	1.037

Supplementary Table S10. Unstandardized estimates, standardized estimates, Z-scores, P-values, and 95% confidence limits (CL, for standardized estimates) for the latent variables of the SEM model for cat owners, Related to STAR Methods. All P-values are corrected for false discovery rate (FDR). Significant (<0.05) P-values are in bold. CL = confidence limit.

Latent variable	Observed variable	Unstand-ardized estimate	Stand-ardized estimate	Z-score	P-value	Lower 95% CL	Upper 95 % CL
Total wellbeing	Life satisfaction	1.000	0.713	28.34	**<0.001**	0.663	0.762
	Perceived stress	-1.362	-0.887	-72.32	**<0.001**	-0.911	-0.863
	Wellbeing	0.802	0.866	69.83	**<0.001**	0.842	0.891
	CESD-10	-1.163	-0.908	-85.61	**<0.001**	-0.929	-0.887
	GAD-7	-0.768	-0.809	-41.74	**<0.001**	-0.847	-0.771

Supplementary Table S11. Unstandardized estimates, standardized estimates, Z-scores, P-values, and 95% confidence limits (CL, for standardized estimates) for intercepts and variances of variables of the SEM model for dog owners, Related to STAR Methods. All P-values are corrected for false discovery rate (FDR). Significant (<0.05) P-values are in bold. CL = confidence limit.

Intercepts

**Variable**	**Unstand-ardized estimate**	**Stand-ardized estimate**	**Z-score**	**P-value**	**Lower 95% CL**	**Upper 95 % CL**
Life satisfaction	22.952	3.894	45.22	**<0.001**	3.725	4.063
Perceived stress	15.199	2.132	78.67	**<0.001**	2.079	2.185
Wellbeing	25.045	5.963	47.05	**<0.001**	5.715	6.212
CESD-10	9.015	1.614	73.44	**<0.001**	1.571	1.658
GAD-7	5.746	1.393	69.96	**<0.001**	1.354	1.433
Dog's fearfulness	0.068	0.063	3.14	**0.003**	0.024	0.103
Dog's barking	0.004	0.004	0.19	0.889	-0.037	0.045
Dog's stranger aggression	0.005	0.005	0.23	0.878	-0.036	0.045
Dog's noise sensitivity	0.032	0.027	1.31	0.245	-0.014	0.068
Dog's separation behavior	-0.013	-0.012	-0.55	0.662	-0.056	0.032
Dog's surface phobia	0.041	0.037	1.74	0.111	-0.005	0.079
Dog's owner aggression	0.004	0.004	0.21	0.880	-0.036	0.045
Dog's dog aggression	-0.018	-0.018	-0.86	0.465	-0.059	0.023
Dog's Vas inattention	-0.003	-0.003	-0.13	0.919	-0.044	0.038
Dog's Vas impulsivity	0.021	0.021	1.01	0.388	-0.020	0.061
PAQ Avoidance	0.650	0.658	3.41	**0.002**	0.280	1.036
PAQ Anxiety	0.377	0.381	2.48	**0.021**	0.080	0.682
Neuroticism	-0.312	-0.287	-1.75	0.109	-0.608	0.034
Agreeableness	0.479	0.683	3.46	**0.002**	0.296	1.070
Conscientiousness	0.898	1.100	21.51	**<0.001**	1.000	1.200
Extraversion	0.137	0.144	6.99	**<0.001**	0.104	0.185
Openness	1.005	1.199	42.94	**<0.001**	1.144	1.253
Owner's gender	1.927	7.403	28.42	**<0.001**	6.893	7.914
Owner's age group	4.973	2.056	NA	NA	2.056	2.056
Children in the household	0.233	0.551	NA	NA	0.551	0.551
Dog's human sociability	0.034	0.035	NA	NA	0.035	0.035
Dog's perseverance	-0.016	-0.016	NA	NA	-0.016	-0.016
Total wellbeng	0.000	0.000	NA	NA	0	0
Fear-aggression	0.000	0.000	NA	NA	0	0
Fear-related behavior	0.000	0.000	NA	NA	0	0
Aggression	0.000	0.000	NA	NA	0	0
Impulsivity/inattention	0.000	0.000	NA	NA	0	0

Variances

Variable	Unstand-ardized estimate	Stand-ardized estimate	Z-score	P-value	Lower 95% CL	Upper 95 % CL
Life satisfaction	18.587	0.535	25.01	**<0.001**	0.493	0.577
Perceived stress	8.929	0.176	15.83	**<0.001**	0.154	0.197
Wellbeing	4.978	0.282	19.05	**<0.001**	0.253	0.311
CESD-10	6.056	0.194	16.92	**<0.001**	0.172	0.217
GAD-7	5.498	0.323	19.33	**<0.001**	0.291	0.356
Dog's fearfulness	0.638	0.549	17.82	**<0.001**	0.488	0.609
Dog's barking	0.014	0.014	0.22	0.880	-0.113	0.140
Dog's stranger aggression	0.444	0.492	14.47	**<0.001**	0.425	0.559
Dog's noise sensitivity	0.953	0.690	17.01	**<0.001**	0.610	0.769
Dog's separation behavior	1.036	0.903	27.19	**<0.001**	0.838	0.968
Dog's surface phobia	0.972	0.817	22.74	**<0.001**	0.746	0.887
Dog's owner aggression	0.737	0.792	19.02	**<0.001**	0.710	0.873
Dog's dog aggression	0.654	0.670	16.42	**<0.001**	0.590	0.750
Dog's Vas inattention	0.579	0.627	14.83	**<0.001**	0.544	0.710
Dog's Vas impulsivity	0.100	0.102	1.19	0.300	-0.067	0.272
PAQ Avoidance	0.847	0.868	43.54	**<0.001**	0.829	0.907
PAQ Anxiety	0.780	0.796	48.97	**<0.001**	0.764	0.828
Neuroticism	1.040	0.880	79.94	**<0.001**	0.858	0.901
Agreeableness	0.479	0.972	127.79	**<0.001**	0.957	0.987
Conscientiousness	0.653	0.980	177.75	**<0.001**	0.969	0.991
Extraversion	0.907	1.000	NA	NA	1.000	1.000
Openness	0.704	1.000	NA	NA	1.000	1.000
Owner's gender	0.068	1.000	NA	NA	1.000	1.000
Owner's age group	5.851	1.000	NA	NA	1.000	1.000
Children in the household	0.179	1.000	NA	NA	1.000	1.000
Dog's human sociability	0.947	1.000	NA	NA	1.000	1.000
Dog's perseverance	0.996	1.000	NA	NA	1.000	1.000
Total wellbeing	7.471	0.463	25.27	**<0.001**	0.427	0.498
Fear-aggression	0.251	1.000	NA	NA	1.000	1.000
Fear-related behavior	0.428	1.000	NA	NA	1.000	1.000
Aggression	0.194	1.000	NA	NA	1.000	1.000
Impulsivity/inattention	0.344	1.000	NA	NA	1.000	1.000

Supplementary Table S12. Unstandardized estimates, standardized estimates, Z-scores, P-values, and 95% confidence limits (CL, for standardized estimates) for intercepts and variances of variables of the SEM model for cat owners, Related to STAR Methods. All P-values are corrected for false discovery rate (FDR). Significant (<0.05) P-values are in bold. CL = confidence limit.

Intercepts

**Variable**	**Unstand-ardized estimate**	**Stand-ardized estimate**	**Z-score**	**P-value**	**Lower 95% CL**	**Upper 95 % CL**
Life satisfaction	22.261	3.439	27.61	**<0.001**	3.195	3.683
Perceived stress	14.945	2.111	43.38	**<0.001**	2.016	2.207
Wellbeing	24.939	5.842	33.25	**<0.001**	5.497	6.186
CESD-10	9.054	1.532	38.89	**<0.001**	1.455	1.609
GAD-7	5.761	1.316	35.77	**<0.001**	1.244	1.389
PAQ Avoidance	0.224	0.225	0.76	0.529	-0.354	0.803
PAQ Anxiety	0.367	0.370	1.52	0.169	-0.107	0.846
Neuroticism	-0.069	-0.063	-0.21	0.881	-0.655	0.529
Agreeableness	0.736	1.027	2.89	**0.007**	0.331	1.723
Conscientiousness	0.574	0.653	7.36	**<0.001**	0.479	0.827
Extraversion	-0.078	-0.083	-2.22	**0.041**	-0.157	-0.010
Openness	1.178	1.414	23.85	**<0.001**	1.297	1.530
Cat's fearfulness	0.124	0.127	3.67	**<0.001**	0.059	0.195
Cat's human aggression	-0.080	-0.093	-2.39	**0.026**	-0.170	-0.017
Cat's activity playfulness	0.003	0.004	0.10	0.940	-0.066	0.074
Cat's cat sociability	0.020	0.022	0.60	0.634	-0.049	0.093
Cat's human sociability	0.015	0.015	0.42	0.751	-0.055	0.086
Cat's litterbox issues	-0.048	-0.052	-1.40	0.208	-0.126	0.021
Cat's excessive grooming	0.005	0.005	0.14	0.919	-0.064	0.074
Owner's gender	1.912	6.766	17.47	**<0.001**	6.007	7.525
Owner's age group	4.659	1.958	NA	NA	1.958	1.958
Children in the household	0.137	0.399	NA	NA	0.399	0.399
Total wellbeing	0.000	0.000	NA	NA	0.000	0.000

Variances

**Variable**	**Unstand-ardized estimate**	**Stand-ardized estimate**	**Z-score**	**P-value**	**Lower 95% CL**	**Upper 95 % CL**
Life satisfaction	20.618	0.492	13.73	**<0.001**	0.422	0.562
Perceived stress	10.657	0.213	9.77	**<0.001**	0.170	0.255
Wellbeing	4.546	0.249	11.60	**<0.001**	0.207	0.292
CESD-10	6.135	0.176	9.12	**<0.001**	0.138	0.213
GAD-7	6.603	0.345	10.98	**<0.001**	0.283	0.406
PAQ Avoidance	0.950	0.956	63.51	**<0.001**	0.926	0.985
PAQ Anxiety	0.635	0.645	20.49	**<0.001**	0.583	0.706
Neuroticism	0.995	0.819	33.85	**<0.001**	0.772	0.867
Agreeableness	0.509	0.991	107.75	**<0.001**	0.973	1.009
Conscientiousness	0.720	0.931	53.09	**<0.001**	0.897	0.966

Extraversion	0.874	1.000	NA	NA	1.000	1.000
Openness	0.695	1.000	NA	NA	1.000	1.000
Cat's fearfulness	0.956	1.000	NA	NA	1.000	1.000
Cat's human aggression	0.729	1.000	NA	NA	1.000	1.000
Cat's activity playfulness	0.931	1.000	NA	NA	1.000	1.000
Cat's cat sociability	0.881	1.000	NA	NA	1.000	1.000
Cat's human sociability	0.951	1.000	NA	NA	1.000	1.000
Cat's litterbox issues	0.853	1.000	NA	NA	1.000	1.000
Cat's excessive grooming	0.978	1.000	NA	NA	1.000	1.000
Owner's gender	0.080	1.000	NA	NA	1.000	1.000
Total wellbeing	9.739	0.458	15.86	**<0.001**	0.401	0.514
Owner's age group	5.664	1.000	NA	NA	1.000	1.000
Children in the household	0.117	1.000	NA	NA	1.000	1.000

Supplementary Table S13. Sample Items of the Dog Personality and Unwanted Behaviour Factors, Related to STAR Methods.

Section	Factor	Sample item
**Personality**	Insecurity	"Insecure: Interested but fearful and uneasy towards new things, vacillates between approach and withdrawal. May need encouragement from people."
	Training focus	“Focused: Is focused on what it is doing despite external stimuli”
	Energy	“Active: Moves about a lot, spends little time idle”
	Aggressiveness/ dominance	“Dominant: Is overbearing and/or threatening towards other dogs”
	Human sociability	“Affectionate (with people): Seeks physical closeness with people. For example, sleeps or relaxes next to people or on their lap, begs petting”
	Dog sociability	“Playful (with dogs): Initiates and engages to play with other dogs”
	Perseverance	“Persevering: Tends to continue in a course of action for a long time, does not give up"
**Unwanted behaviour**	Noise sensitivity	“When hearing fireworks, my dog escapes”.
	Fearfulness	“When meeting a stranger my dog withdraws”
	Fear of surfaces or hights	“Does your dog have difficulties to walk on a metal grid?”
	Separation anxiety	“The dog pants when the owner is leaving”
	Impulsivity	“My dog does not think before she/he acts (e.g., would steal food without first looking to see if someone is watching)”
	Inattention	“It's difficult for my dog to concentrate on a task or play.”
	Barking	“The dog barks when the doorbell rings or the door is knocked”
	Stanger directed aggression	“When a stranger tries to pet the dog outside the home and the dog is on a leash, my dog tries to snap or bite the stranger”
	Owner directed aggression	“When the owner/family member takes a bone/food/toy from the dog, she/he tries to snap or bite”
	Dog directed aggression	“When my dog meets an unfamiliar dog, my dog tries to attack it”

Supplementary Table S14. Sample Items of the Cat Personality and Unwanted Behaviour Factors, Related to STAR Methods.

Section	Factor	Sample item
Personality	Fearfulness	"Escapes or hides from unfamiliar people"
	Activity/ playfulness	"Stalks, chases, or pounces on moving objects (e.g., string, balls, soft toys)"
	Aggression toward humans	"Attempts to scratch or bite when his/her nails are clipped"
	Sociability toward humans	"Often seeks out physical contact from people, nudges or nuzzles"
	Sociability toward cats	"Seeks company of other cats in the household"
Unwanted behaviour	Excessive grooming	"Shows excessive and intensive grooming (inhibits other behaviours) throughout the day"
	Litterbox issues	"Urinates (crouching position) in inappropriate places"

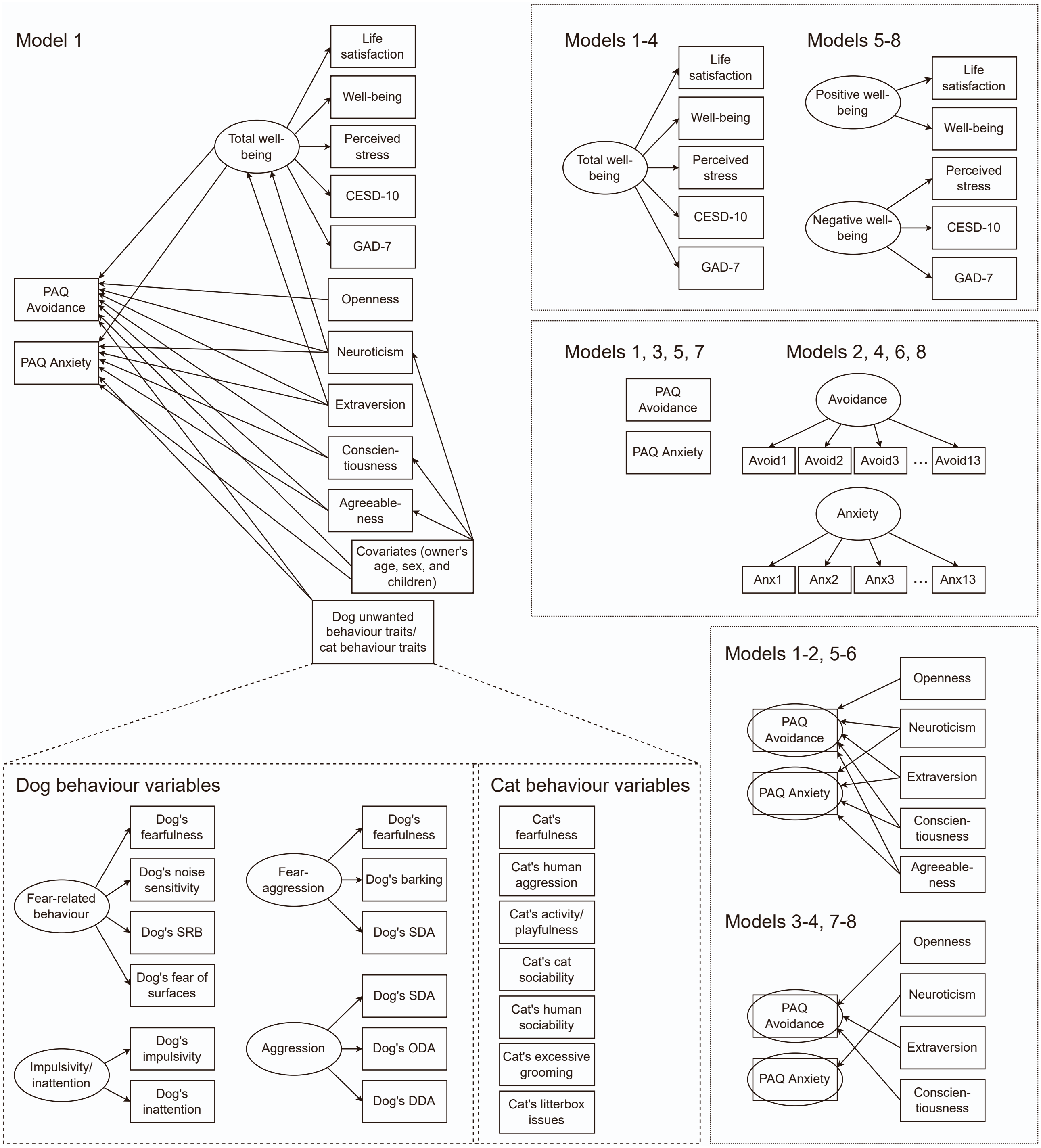

Supplementary Figure S1. Competing models, Related to STAR Methods. Regressions and latent variables of model 1 are shown as an example. Models 1-4 included one latent well-being variable (total well-being) and models 5-8 included two latent well-being variables (positive well-being and negative well-being). Models 1, 3, 5, and 7 utilized original pet attachment scores and models 2, 4, 6, and 8 utilized latent attachment variables. Models 1-2 and 5-6 included more regressions from owner personality traits to attachment scales/latent variables, and models 3-4 and 7-8 included fewer regressions. Competing models were identical in dog and cat owners, except those models of dog owners included latent unwanted dog behavioural traits and models of cat owners included cat behavioural traits.

Supplemental references

1. [S2] Zilcha-Mano, S., Mikulincer, M., & Shaver, P. R. (2011). An attachment perspective on human–pet relationships: Conceptualization and assessment of pet attachment orientations. *Journal of Research in Personality*, *45*(4), 345-357. 10.1016/j.jrp.2011.04.001.
