# Supplementary material for: Pet and owner personality and mental wellbeing associate with attachment to cats and dogs
Source: iScience. 2023 Nov 9;26(12):108423. doi: 10.1016/j.isci.2023.108423 (PMC10709106; doi:10.1016/j.isci.2023.108423)
Supplement: Data S1. R-code [file mmc2.pdf]

Supplementary information: R code

Aada Ståhl, Milla Salonen, Emma Hakanen, Salla Mikkola, Sini Sulkama, Jari Lahti, Hannes Lohi

```
#### PACKAGES ####

library(dplyr)
library(lavaan)
library(semTools)



#### DATA EDITS ####

#first read in data

onep.paq <- read.table()

# owner's gender
levels(onep.paq$owner_gender) <- c("not_woman","not_woman","not_woman","woman")
levels(onep.paq$owner_gender) <- c(1,2)
onep.paq$owner_gender <- as.numeric(as.character(onep.paq$owner_gender))

# age
levels(onep.paq$owner_age) <- c(2,3,4,5,6,7,8,9,1,NA)

# children
levels(onep.paq$children) <- c(0,1)
onep.paq$children <- as.numeric(as.character(onep.paq$children))

# scale PAQ traits as contain much more variance than other variables
onep.paq$PAQ_Anxiety_scaled <- scale(onep.paq$PAQ_Anxiety)
onep.paq$PAQ_Avoidance_scaled <- scale(onep.paq$PAQ_Avoidance)



#### DOGS ####

# filter dogs
paq.dogs <- filter(onep.paq, species=="dog")
paq.dogs <- droplevels(paq.dogs)


#### ** Model selection ####


#### **** Dog model 1: Total wellbeing, scales, large personality ####

paq.sem.dog.1 <- '
    # latent variables
    Total_wellbeing =~ Life.satisfaction + Perceived.stress + Wellbeing +
                CESD.10 + GAD.7
```

```
    fearaggre =~ DOG_fearfulness + DOG_barking + DOG_stranger_aggression
    fear.related =~ DOG_noise_sensitivity + DOG_separation_behavior +
                     DOG_surface_phobia + DOG_fearfulness
    aggression =~ DOG_owner_aggression + DOG_dog_aggression + DOG_stranger_aggression
    adhd =~ DOG_Vas_inattention + DOG_Vas_impulsivity

    # regressions
    PAQ_Avoidance_scaled ~ Agreeableness2M + ConscientiousnessM + ExtraversionM +
                          NeuroticismM + OpennessM +
                          fearaggre + aggression + adhd +
                          owner_gender + owner_age_num +
                          children + Total_wellbeing
    PAQ_Anxiety_scaled ~ Agreeableness2M + ConscientiousnessM + ExtraversionM +
                          NeuroticismM +
                           fearaggre + fear.related +
                           owner_gender + owner_age_num +
                          children + Total_wellbeing
    Total_wellbeing ~ NeuroticismM + ExtraversionM
    NeuroticismM ~ owner_gender + owner_age_num
    Agreeableness2M ~ owner_gender + owner_age_num
    ConscientiousnessM ~ owner_age_num

    # correlations
    Total_wellbeing ~~ owner_gender
    PAQ_Avoidance_scaled ~~ 0*PAQ_Anxiety_scaled
    fearaggre ~~ aggression
    fear.related ~~ adhd
    NeuroticismM ~~ ConscientiousnessM
    NeuroticismM ~~ ExtraversionM
    OpennessM ~~ ExtraversionM
    ExtraversionM ~~ ConscientiousnessM
    Agreeableness2M ~~ NeuroticismM + OpennessM
'

# run the model
fit.paq.sem.dog.1 <- sem(paq.sem.dog.1, estimator="MLR", missing="ml.x", fixed.x=T, data =
paq.dogs)

# extract model fit measures
totwell.scale.large.dog <- fitmeasures(fit.paq.sem.dog.1, c("cfi","tli","rmsea","srmr"))




#### **** Dog model 2: Total wellbeing, latent paq, large personality ####


paq.sem.dog.2 <- '
    # latent variables
    Anxiety =~ Anx1 + Anx2 + Anx3 + Anx4 + Anx5 + Anx6 + Anx7 + Anx8 + Anx9 + Anx10 +
Anx11 + Anx12 + Anx13
    Avoidance =~ Avoid1 + Avoid2 + Avoid3 + Avoid4 + Avoid5 + Avoid6 + Avoid7 + Avoid8 +
                Avoid9 + Avoid10 + Avoid11 + Avoid12 + Avoid13
```

```
    Total_wellbeing =~ Life.satisfaction + Perceived.stress + Wellbeing +
                CESD.10 + GAD.7
    fearaggre =~ DOG_fearfulness + DOG_barking + DOG_stranger_aggression
    fear.related =~ DOG_noise_sensitivity + DOG_separation_behavior +
                      DOG_surface_phobia + DOG_fearfulness
    aggression =~ DOG_owner_aggression + DOG_dog_aggression + DOG_stranger_aggression
    adhd =~ DOG_Vas_inattention + DOG_Vas_impulsivity

    # regressions
    Avoidance ~ Agreeableness2M + ConscientiousnessM + ExtraversionM +
                               NeuroticismM + OpennessM +
                               fearaggre + aggression + adhd +
                               owner_gender + owner_age_num +
                               children + Total_wellbeing
    Anxiety ~ Agreeableness2M + ConscientiousnessM + ExtraversionM +
                               NeuroticismM +
                                fearaggre + fear.related +
                                owner_gender + owner_age_num +
                               children + Total_wellbeing
    Total_wellbeing ~ NeuroticismM + ExtraversionM
    NeuroticismM ~ owner_gender + owner_age_num
    Agreeableness2M ~ owner_gender + owner_age_num
    ConscientiousnessM ~ owner_age_num

    # correlations
    Total_wellbeing ~~ owner_gender
    Avoidance ~~ 0*Anxiety
    fearaggre ~~ aggression
    fear.related ~~ adhd
    NeuroticismM ~~ ConscientiousnessM
    NeuroticismM ~~ ExtraversionM
    OpennessM ~~ ExtraversionM
    ExtraversionM ~~ ConscientiousnessM
    Agreeableness2M ~~ NeuroticismM + OpennessM
'

fit.paq.sem.dog.2 <- sem(paq.sem.dog.2, estimator="MLR", missing="ml.x", fixed.x=T, data =
paq.dogs)
totwell.latent.large.dog <- fitmeasures(fit.paq.sem.dog.2, c("cfi","tli","rmsea","srmr"))



#### **** Dog model 3: Total wellbeing, scales, small personality ####

paq.sem.dog.3 <- '
    # latent variables
    Total_wellbeing =~ Life.satisfaction + Perceived.stress + Wellbeing +
                CESD.10 + GAD.7
    fearaggre =~ DOG_fearfulness + DOG_barking + DOG_stranger_aggression
    fear.related =~ DOG_noise_sensitivity + DOG_separation_behavior +
                      DOG_surface_phobia + DOG_fearfulness
    aggression =~ DOG_owner_aggression + DOG_dog_aggression + DOG_stranger_aggression
    adhd =~ DOG_Vas_inattention + DOG_Vas_impulsivity

    # regressions
```

```
    PAQ_Avoidance_scaled ~ ConscientiousnessM + ExtraversionM +
                              OpennessM  +
                              fearaggre + aggression + adhd +
                              owner_gender + owner_age_num +
                              children + Total_wellbeing
    PAQ_Anxiety_scaled ~      NeuroticismM +
                               fearaggre + fear.related +
                               owner_gender + owner_age_num +
                              children + Total_wellbeing
    Total_wellbeing ~ NeuroticismM + ExtraversionM
    NeuroticismM ~ owner_gender + owner_age_num
    ConscientiousnessM ~ owner_age_num

    # correlations
    Total_wellbeing ~~ owner_gender
    PAQ_Avoidance_scaled ~~ 0*PAQ_Anxiety_scaled
    fearaggre ~~ aggression
    fear.related ~~ adhd
    NeuroticismM ~~ ConscientiousnessM
    NeuroticismM ~~ ExtraversionM
    OpennessM ~~ ExtraversionM
    ExtraversionM ~~ ConscientiousnessM
    Agreeableness2M ~~ NeuroticismM + OpennessM
'

fit.paq.sem.dog.3 <- sem(paq.sem.dog.3, estimator="MLR", missing="ml.x", fixed.x=T, data =
paq.dogs)
totwell.scale.small.dog <- fitmeasures(fit.paq.sem.dog.3, c("cfi","tli","rmsea","srmr"))



#### **** Dog model 4: Total wellbeing, latent paq, small personality ####


paq.sem.dog.4 <- '
    # latent variables
    Total_wellbeing =~ Life.satisfaction + Perceived.stress + Wellbeing +
                CESD.10 + GAD.7
    Anxiety =~ Anx1 + Anx2 + Anx3 + Anx4 + Anx5 + Anx6 + Anx7 + Anx8 + Anx9 + Anx10 +
Anx11 + Anx12 + Anx13
    Avoidance =~ Avoid1 + Avoid2 + Avoid3 + Avoid4 + Avoid5 + Avoid6 + Avoid7 + Avoid8 +
                Avoid9 + Avoid10 + Avoid11 + Avoid12 + Avoid13
    fearaggre =~ DOG_fearfulness + DOG_barking + DOG_stranger_aggression
    fear.related =~ DOG_noise_sensitivity + DOG_separation_behavior +
                  DOG_surface_phobia + DOG_fearfulness
    aggression =~ DOG_owner_aggression + DOG_dog_aggression + DOG_stranger_aggression
    adhd =~ DOG_Vas_inattention + DOG_Vas_impulsivity

    # regressions
    Avoidance ~ ConscientiousnessM + ExtraversionM +
                              OpennessM +
                              fearaggre + aggression + adhd +
                              owner_gender + owner_age_num +
                              children + Total_wellbeing
    Anxiety ~      NeuroticismM +
```

```
                               fearaggre + fear.related +
                               owner_gender + owner_age_num +
                              children + Total_wellbeing
    Total_wellbeing ~ NeuroticismM + ExtraversionM
    NeuroticismM ~ owner_gender + owner_age_num
    ConscientiousnessM ~ owner_age_num

    # correlations
    Total_wellbeing ~~ owner_gender
    Avoidance ~~ 0*Anxiety
    fearaggre ~~ aggression
    fear.related ~~ adhd
    NeuroticismM ~~ ConscientiousnessM
    NeuroticismM ~~ ExtraversionM
    OpennessM ~~ ExtraversionM
    ExtraversionM ~~ ConscientiousnessM
    Agreeableness2M ~~ NeuroticismM + OpennessM
'

fit.paq.sem.dog.4 <- sem(paq.sem.dog.4, estimator="MLR", missing="ml.x", fixed.x=T, data =
paq.dogs)
totwell.latent.small.dog <- fitmeasures(fit.paq.sem.dog.4, c("cfi","tli","rmsea","srmr"))



#### **** Dog model 5: Positive & negative wellbeing, scales, large personality ####

paq.sem.dog.5 <- '
    # latent variables
    Positive_well =~ Life.satisfaction + Wellbeing
    Negative_well =~ Perceived.stress + CESD.10 + GAD.7
    fearaggre =~ DOG_fearfulness + DOG_barking + DOG_stranger_aggression
    fear.related =~ DOG_noise_sensitivity + DOG_separation_behavior +
                      DOG_surface_phobia + DOG_fearfulness
    aggression =~ DOG_owner_aggression + DOG_dog_aggression + DOG_stranger_aggression
    adhd =~ DOG_Vas_inattention + DOG_Vas_impulsivity

    # regressions
    PAQ_Avoidance_scaled ~ Agreeableness2M + ConscientiousnessM + ExtraversionM +
                              NeuroticismM + OpennessM +
                              fearaggre + aggression + adhd +
                              owner_gender + owner_age_num +
                              children + Positive_well + Negative_well
    PAQ_Anxiety_scaled ~ Agreeableness2M + ConscientiousnessM + ExtraversionM +
                              NeuroticismM +
                               fearaggre + fear.related +
                               owner_gender + owner_age_num +
                              children + Positive_well + Negative_well
    Positive_well ~ NeuroticismM + ExtraversionM
    Negative_well ~ NeuroticismM + ExtraversionM
    NeuroticismM ~ owner_gender + owner_age_num
    Agreeableness2M ~ owner_gender + owner_age_num
    ConscientiousnessM ~ owner_age_num

    # correlations
```

```
    Positive_well ~~ owner_gender
    Negative_well ~~ owner_gender
    PAQ_Avoidance_scaled ~~ 0*PAQ_Anxiety_scaled
    fearaggre ~~ aggression
    fear.related ~~ adhd
    NeuroticismM ~~ ConscientiousnessM
    NeuroticismM ~~ ExtraversionM
    OpennessM ~~ ExtraversionM
    ExtraversionM ~~ ConscientiousnessM
    Agreeableness2M ~~ NeuroticismM + OpennessM
'

fit.paq.sem.dog.5 <- sem(paq.sem.dog.5, estimator="MLR", missing="ml.x", fixed.x=T, data =
paq.dogs)
posinegawell.scale.large.dog <- fitmeasures(fit.paq.sem.dog.5,
c("cfi","tli","rmsea","srmr"))




#### **** Dog model 6: Positive & negative wellbeing, latent paq, large personality ####


paq.sem.dog.6 <- '
    # latent variables
    Anxiety =~ Anx1 + Anx2 + Anx3 + Anx4 + Anx5 + Anx6 + Anx7 + Anx8 + Anx9 + Anx10 +
Anx11 + Anx12 + Anx13
    Avoidance =~ Avoid1 + Avoid2 + Avoid3 + Avoid4 + Avoid5 + Avoid6 + Avoid7 + Avoid8 +
                    Avoid9 + Avoid10 + Avoid11 + Avoid12 + Avoid13

    Positive_well =~ Life.satisfaction + Wellbeing
    Negative_well =~ Perceived.stress + CESD.10 + GAD.7
    fearaggre =~ DOG_fearfulness + DOG_barking + DOG_stranger_aggression
    fear.related =~ DOG_noise_sensitivity + DOG_separation_behavior +
                      DOG_surface_phobia + DOG_fearfulness
    aggression =~ DOG_owner_aggression + DOG_dog_aggression + DOG_stranger_aggression
    adhd =~ DOG_Vas_inattention + DOG_Vas_impulsivity

    # regressions
    Avoidance ~ Agreeableness2M + ConscientiousnessM + ExtraversionM +
                          NeuroticismM + OpennessM +
                          fearaggre + aggression + adhd +
                          owner_gender + owner_age_num +
                          children + Positive_well + Negative_well
    Anxiety ~ Agreeableness2M + ConscientiousnessM + ExtraversionM +
                          NeuroticismM +
                           fearaggre + fear.related +
                           owner_gender + owner_age_num +
                          children + Positive_well + Negative_well
    Positive_well ~ NeuroticismM + ExtraversionM
    Negative_well ~ NeuroticismM + ExtraversionM
    NeuroticismM ~ owner_gender + owner_age_num
    Agreeableness2M ~ owner_gender + owner_age_num
    ConscientiousnessM ~ owner_age_num
```

```
    # correlations
    Positive_well ~~ owner_gender
    Negative_well ~~ owner_gender
    Avoidance ~~ 0*Anxiety
    fearaggre ~~ aggression
    fear.related ~~ adhd
    NeuroticismM ~~ ConscientiousnessM
    NeuroticismM ~~ ExtraversionM
    OpennessM ~~ ExtraversionM
    ExtraversionM ~~ ConscientiousnessM
    Agreeableness2M ~~ NeuroticismM + OpennessM
'

fit.paq.sem.dog.6 <- sem(paq.sem.dog.6, estimator="MLR", missing="ml.x", fixed.x=T, data =
paq.dogs)
posinegawell.latent.large.dog <- fitmeasures(fit.paq.sem.dog.6,
c("cfi","tli","rmsea","srmr"))




#### **** Dog model 7: Positive & negative wellbeing, scales, small personality ####

paq.sem.dog.7 <- '
    # latent variables
    Positive_well =~ Life.satisfaction + Wellbeing
    Negative_well =~ Perceived.stress + CESD.10 + GAD.7
    fearaggre =~ DOG_fearfulness + DOG_barking + DOG_stranger_aggression
    fear.related =~ DOG_noise_sensitivity + DOG_separation_behavior +
                      DOG_surface_phobia + DOG_fearfulness
    aggression =~ DOG_owner_aggression + DOG_dog_aggression + DOG_stranger_aggression
    adhd =~ DOG_Vas_inattention + DOG_Vas_impulsivity

    # regressions
    PAQ_Avoidance_scaled ~ ConscientiousnessM + ExtraversionM +
                              OpennessM  +
                              fearaggre + aggression + adhd +
                              owner_gender + owner_age_num +
                              children + Positive_well + Negative_well
    PAQ_Anxiety_scaled ~      NeuroticismM +
                               fearaggre + fear.related +
                               owner_gender + owner_age_num +
                              children + Positive_well + Negative_well
    Positive_well ~ NeuroticismM + ExtraversionM
    Negative_well ~ NeuroticismM + ExtraversionM
    NeuroticismM ~ owner_gender + owner_age_num
    ConscientiousnessM ~ owner_age_num

    # correlations
    Positive_well ~~ owner_gender
    Negative_well ~~ owner_gender
    PAQ_Avoidance_scaled ~~ 0*PAQ_Anxiety_scaled
    fearaggre ~~ aggression
    fear.related ~~ adhd
    NeuroticismM ~~ ConscientiousnessM
```

```
    NeuroticismM ~~ ExtraversionM
    OpennessM ~~ ExtraversionM
    ExtraversionM ~~ ConscientiousnessM
    Agreeableness2M ~~ NeuroticismM + OpennessM
'

fit.paq.sem.dog.7 <- sem(paq.sem.dog.7, estimator="MLR", missing="ml.x", fixed.x=T, data =
paq.dogs)
posinegawell.scale.small.dog <- fitmeasures(fit.paq.sem.dog.7,
c("cfi","tli","rmsea","srmr"))




#### **** Dog model 8: Positive & negative wellbeing, latent paq, small personality ####


paq.sem.dog.8 <- '
    # latent variables
    Positive_well =~ Life.satisfaction + Wellbeing
    Negative_well =~ Perceived.stress + CESD.10 + GAD.7
    Anxiety =~ Anx1 + Anx2 + Anx3 + Anx4 + Anx5 + Anx6 + Anx7 + Anx8 + Anx9 + Anx10 +
Anx11 + Anx12 + Anx13
    Avoidance =~ Avoid1 + Avoid2 + Avoid3 + Avoid4 + Avoid5 + Avoid6 + Avoid7 + Avoid8 +
                Avoid9 + Avoid10 + Avoid11 + Avoid12 + Avoid13
    fearaggre =~ DOG_fearfulness + DOG_barking + DOG_stranger_aggression
    fear.related =~ DOG_noise_sensitivity + DOG_separation_behavior +
                  DOG_surface_phobia + DOG_fearfulness
    aggression =~ DOG_owner_aggression + DOG_dog_aggression + DOG_stranger_aggression
    adhd =~ DOG_Vas_inattention + DOG_Vas_impulsivity

    # regressions
    Avoidance ~ ConscientiousnessM + ExtraversionM +
                          OpennessM +
                          fearaggre + aggression + adhd +
                          owner_gender + owner_age_num +
                          children + Positive_well + Negative_well
    Anxiety ~      NeuroticismM +
                           fearaggre + fear.related +
                           owner_gender + owner_age_num +
                          children + Positive_well + Negative_well
    Positive_well ~ NeuroticismM + ExtraversionM
    Negative_well ~ NeuroticismM + ExtraversionM
    NeuroticismM ~ owner_gender + owner_age_num
    ConscientiousnessM ~ owner_age_num

    # correlations
    Positive_well ~~ owner_gender
    Negative_well ~~ owner_gender
    Avoidance ~~ 0*Anxiety
    fearaggre ~~ aggression
    fear.related ~~ adhd
    NeuroticismM ~~ ConscientiousnessM
    NeuroticismM ~~ ExtraversionM
    OpennessM ~~ ExtraversionM
```

```
    ExtraversionM ~~ ConscientiousnessM
    Agreeableness2M ~~ NeuroticismM + OpennessM
'

fit.paq.sem.dog.8 <- sem(paq.sem.dog.8, estimator="MLR", missing="ml.x", fixed.x=T, data =
paq.dogs)
# vcov not positive definite
# cfi = 0.741, tli = 0.722, rmsea = 0.057, srmr = 0.075
posinegawell.latent.small.dog <- fitmeasures(fit.paq.sem.dog.8,
c("cfi","tli","rmsea","srmr"))



#### ** Model fit comparison ####

rbind(totwell.scale.large.dog,totwell.latent.large.dog,
      totwell.scale.small.dog,totwell.latent.small.dog,
      posinegawell.scale.large.dog,posinegawell.latent.large.dog,
      posinegawell.scale.small.dog,posinegawell.latent.small.dog)

# cfi: 1&3 equal, 5&7 pretty close
# tli: 1&3 equal, 5&7 pretty close
# rmsea: 1&3 equal, 2&4 kinda close
# srmr: 1 best, 3 then, 2 next

### FIRST OVERALL BEST


#### ** Final dog model: 1st model with human sociability and perseverance ####

paq.sem.dog.final <- '
    # latent variables
    Total_wellbeing =~ Life.satisfaction + Perceived.stress + Wellbeing +
                CESD.10 + GAD.7
    fearaggre =~ DOG_fearfulness + DOG_barking + DOG_stranger_aggression
    fear.related =~ DOG_noise_sensitivity + DOG_separation_behavior +
                  DOG_surface_phobia + DOG_fearfulness
    aggression =~ DOG_owner_aggression + DOG_dog_aggression + DOG_stranger_aggression
    adhd =~ DOG_Vas_inattention + DOG_Vas_impulsivity

    # regressions
    PAQ_Avoidance_scaled ~ Agreeableness2M + ConscientiousnessM + ExtraversionM +
                              NeuroticismM + OpennessM +
                              fearaggre + aggression + adhd +
                              owner_gender + owner_age_num +
                              children + Total_wellbeing + DOG_human_sociability
    PAQ_Anxiety_scaled ~ Agreeableness2M + ConscientiousnessM + ExtraversionM +
                              NeuroticismM +
                               fearaggre + fear.related +
                               owner_gender + owner_age_num +
                              children + Total_wellbeing + DOG_perseverance
    Total_wellbeing ~ NeuroticismM + ExtraversionM
    NeuroticismM ~ owner_gender + owner_age_num
    Agreeableness2M ~ owner_gender + owner_age_num
```

```
    ConscientiousnessM ~ owner_age_num

    # correlations
    Total_wellbeing ~~ owner_gender
    PAQ_Avoidance_scaled ~~ 0*PAQ_Anxiety_scaled
    fearaggre ~~ aggression
    fear.related ~~ adhd
    NeuroticismM ~~ ConscientiousnessM
    NeuroticismM ~~ ExtraversionM
    OpennessM ~~ ExtraversionM
    ExtraversionM ~~ ConscientiousnessM
    Agreeableness2M ~~ NeuroticismM + OpennessM
'

# run the model
fit.paq.sem.dog.final <- sem(paq.sem.dog.final, estimator="MLR", missing="ml.x",
fixed.x=T, data = paq.dogs)

# extract model fit measures
final.dog.model <- fitmeasures(fit.paq.sem.dog.final, c("cfi","tli","rmsea","srmr"))

# obtain the standardized and unstandardized estimates
standardizedSolution(fit.paq.sem.dog.final , type="std.all", output="pretty")
parameterEstimates(fit.paq.sem.dog.final)


#### ** RMSEA of null model ####

nullRMSEA(fit.paq.sem.dog.final)

# "A reasonable rule of thumb is to examine the RMSEA for the null model and make sure
that is no
# smaller than 0.158. An RMSEA for the model of 0.05 and a TLI of .90, implies that the
RMSEA
# of the null model is 0.158. If the RMSEA for the null model is less than 0.158, an
incremental
# measure of fit may not be that informative."
# See also http://davidakenny.net/cm/fit.htm




#### CATS ####

# filter cats
paq.cats <- filter(onep.paq, species=="cat")
paq.cats <- droplevels(paq.cats)



#### ** Model selection ####


#### **** Cat model 1: Total wellbeing, scales, large personality ####
```

```
cats.sem.1 <- '
    # latent variables
    Total_wellbeing =~ Life.satisfaction + Perceived.stress + Wellbeing +
                CESD.10 + GAD.7

    # regressions
    PAQ_Avoidance_scaled ~ Agreeableness2M + ConscientiousnessM + ExtraversionM +
                              NeuroticismM + OpennessM +
                              CAT_fearfulness + CAT_human_aggression +
                              CAT_activity_playfulness + CAT_cat_sociability +
                              CAT_human_sociability + CAT_litterbox_issues +
                              CAT_excessive_grooming +
                              owner_gender + owner_age_num +
                              children + Total_wellbeing
    PAQ_Anxiety_scaled ~ Agreeableness2M + ConscientiousnessM + ExtraversionM +
                              NeuroticismM +
                              CAT_fearfulness + CAT_human_aggression +
                              CAT_activity_playfulness + CAT_cat_sociability +
                              CAT_human_sociability + CAT_litterbox_issues +
                              CAT_excessive_grooming +
                              owner_gender + owner_age_num +
                              children + Total_wellbeing
    Total_wellbeing ~ NeuroticismM + ExtraversionM
    NeuroticismM ~ owner_gender + owner_age_num
    Agreeableness2M ~ owner_gender + owner_age_num
    ConscientiousnessM ~ owner_age_num

    # correlations
    Total_wellbeing ~~ owner_gender
    PAQ_Avoidance_scaled ~~ 0*PAQ_Anxiety_scaled
    NeuroticismM ~~ ConscientiousnessM
    NeuroticismM ~~ ExtraversionM
    OpennessM ~~ ExtraversionM
    ExtraversionM ~~ ConscientiousnessM
    Agreeableness2M ~~ NeuroticismM + OpennessM
    CAT_cat_sociability ~~ CAT_activity_playfulness + CAT_human_aggression +
CAT_litterbox_issues
    CAT_human_sociability ~~ CAT_activity_playfulness + CAT_fearfulness
    CAT_human_aggression ~~ CAT_fearfulness
    CAT_litterbox_issues ~~ CAT_excessive_grooming
'

fit.cat.sem.1 <- sem(cats.sem.1, estimator="MLR", missing="ml.x", fixed.x=T, data =
paq.cats)
totwell.scale.large.cat <- fitmeasures(fit.cat.sem.1, c("cfi","tli","rmsea","srmr"))




#### **** Cat model 2: Total wellbeing, latent paq, large personality ####


cats.sem.2 <- '
    # latent variables
```

```
    Total_wellbeing =~ Life.satisfaction + Perceived.stress + Wellbeing +
                CESD.10 + GAD.7
    Anxiety =~ Anx1 + Anx2 + Anx3 + Anx4 + Anx5 + Anx6 + Anx7 + Anx8 + Anx9 + Anx10 +
Anx11 + Anx12 + Anx13
    Avoidance =~ Avoid1 + Avoid2 + Avoid3 + Avoid4 + Avoid5 + Avoid6 + Avoid7 + Avoid8 +
                    Avoid9 + Avoid10 + Avoid11 + Avoid12 + Avoid13

    # regressions
    Avoidance ~ Agreeableness2M + ConscientiousnessM + ExtraversionM +
                          NeuroticismM + OpennessM +
                          CAT_fearfulness + CAT_human_aggression +
                          CAT_activity_playfulness + CAT_cat_sociability +
                          CAT_human_sociability + CAT_litterbox_issues +
                          CAT_excessive_grooming +
                          owner_gender + owner_age_num +
                          children + Total_wellbeing
    Anxiety ~ Agreeableness2M + ConscientiousnessM + ExtraversionM +
                          NeuroticismM +
                          CAT_fearfulness + CAT_human_aggression +
                          CAT_activity_playfulness + CAT_cat_sociability +
                          CAT_human_sociability + CAT_litterbox_issues +
                          CAT_excessive_grooming +
                          owner_gender + owner_age_num +
                          children + Total_wellbeing
    Total_wellbeing ~ NeuroticismM + ExtraversionM
    NeuroticismM ~ owner_gender + owner_age_num
    Agreeableness2M ~ owner_gender + owner_age_num
    ConscientiousnessM ~ owner_age_num

    # correlations
    Total_wellbeing ~~ owner_gender
    Avoidance ~~ 0*Anxiety
    NeuroticismM ~~ ConscientiousnessM
    NeuroticismM ~~ ExtraversionM
    OpennessM ~~ ExtraversionM
    ExtraversionM ~~ ConscientiousnessM
    Agreeableness2M ~~ NeuroticismM + OpennessM
    CAT_cat_sociability ~~ CAT_activity_playfulness + CAT_human_aggression +
CAT_litterbox_issues
    CAT_human_sociability ~~ CAT_activity_playfulness + CAT_fearfulness
    CAT_human_aggression ~~ CAT_fearfulness
    CAT_litterbox_issues ~~ CAT_excessive_grooming
'

fit.cat.sem.2 <- sem(cats.sem.2, estimator="MLR", missing="ml.x", fixed.x=T, data =
paq.cats)
totwell.latent.large.cat <- fitmeasures(fit.cat.sem.2, c("cfi","tli","rmsea","srmr"))




#### **** Cat model 3: Total wellbeing, scales, small personality ####

cats.sem.3 <- '
    # latent variables
```

```
    Total_wellbeing =~ Life.satisfaction + Perceived.stress + Wellbeing +
                CESD.10 + GAD.7

    # regressions
    PAQ_Avoidance_scaled ~ ConscientiousnessM + ExtraversionM +
                           OpennessM +
                           CAT_fearfulness + CAT_human_aggression +
                           CAT_activity_playfulness + CAT_cat_sociability +
                           CAT_human_sociability + CAT_litterbox_issues +
                           CAT_excessive_grooming +
                           owner_gender + owner_age_num +
                           children + Total_wellbeing
    PAQ_Anxiety_scaled ~ NeuroticismM +
                           CAT_fearfulness + CAT_human_aggression +
                           CAT_activity_playfulness + CAT_cat_sociability +
                           CAT_human_sociability + CAT_litterbox_issues +
                           CAT_excessive_grooming +
                           owner_gender + owner_age_num +
                           children + Total_wellbeing
    Total_wellbeing ~ NeuroticismM + ExtraversionM
    NeuroticismM ~ owner_gender + owner_age_num
    ConscientiousnessM ~ owner_age_num

    # correlations
    Total_wellbeing ~~ owner_gender
    PAQ_Avoidance_scaled ~~ 0*PAQ_Anxiety_scaled
    NeuroticismM ~~ ConscientiousnessM
    NeuroticismM ~~ ExtraversionM
    OpennessM ~~ ExtraversionM
    ExtraversionM ~~ ConscientiousnessM
    CAT_cat_sociability ~~ CAT_activity_playfulness + CAT_human_aggression +
CAT_litterbox_issues
    CAT_human_sociability ~~ CAT_activity_playfulness + CAT_fearfulness
    CAT_human_aggression ~~ CAT_fearfulness
    CAT_litterbox_issues ~~ CAT_excessive_grooming
'

fit.cat.sem.3 <- sem(cats.sem.3, estimator="MLR", missing="ml.x", fixed.x=T, data =
paq.cats)
totwell.scale.small.cat <- fitmeasures(fit.cat.sem.3, c("cfi","tli","rmsea","srmr"))



#### **** Cat model 4: Total wellbeing, latent paq, small personality ####


cats.sem.4 <- '
    # latent variables
    Total_wellbeing =~ Life.satisfaction + Perceived.stress + Wellbeing +
                CESD.10 + GAD.7
    Anxiety =~ Anx1 + Anx2 + Anx3 + Anx4 + Anx5 + Anx6 + Anx7 + Anx8 + Anx9 + Anx10 +
Anx11 + Anx12 + Anx13
    Avoidance =~ Avoid1 + Avoid2 + Avoid3 + Avoid4 + Avoid5 + Avoid6 + Avoid7 + Avoid8 +
                    Avoid9 + Avoid10 + Avoid11 + Avoid12 + Avoid13
```

```
    # regressions
    Avoidance ~ ConscientiousnessM + ExtraversionM +
                              OpennessM +
                              CAT_fearfulness + CAT_human_aggression +
                              CAT_activity_playfulness + CAT_cat_sociability +
                              CAT_human_sociability + CAT_litterbox_issues +
                              CAT_excessive_grooming +
                              owner_gender + owner_age_num +
                              children + Total_wellbeing
    Anxiety ~                 NeuroticismM +
                              CAT_fearfulness + CAT_human_aggression +
                              CAT_activity_playfulness + CAT_cat_sociability +
                              CAT_human_sociability + CAT_litterbox_issues +
                              CAT_excessive_grooming +
                              owner_gender + owner_age_num +
                              children + Total_wellbeing
    Total_wellbeing ~ NeuroticismM + ExtraversionM
    NeuroticismM ~ owner_gender + owner_age_num
    ConscientiousnessM ~ owner_age_num

    # correlations
    Total_wellbeing ~~ owner_gender
    Avoidance ~~ 0*Anxiety
    NeuroticismM ~~ ConscientiousnessM
    NeuroticismM ~~ ExtraversionM
    OpennessM ~~ ExtraversionM
    ExtraversionM ~~ ConscientiousnessM
    CAT_cat_sociability ~~ CAT_activity_playfulness + CAT_human_aggression +
CAT_litterbox_issues
    CAT_human_sociability ~~ CAT_activity_playfulness + CAT_fearfulness
    CAT_human_aggression ~~ CAT_fearfulness
    CAT_litterbox_issues ~~ CAT_excessive_grooming
'

fit.cat.sem.4 <- sem(cats.sem.4, estimator="MLR", missing="ml.x", fixed.x=T, data =
paq.cats)
totwell.latent.small.cat <- fitmeasures(fit.cat.sem.4, c("cfi","tli","rmsea","srmr"))




#### **** Cat model 5: Positive & negative wellbeing, scales, large personality ####


cats.sem.5 <- '
    # latent variables
    Positive_well =~ Life.satisfaction + Wellbeing
    Negative_well =~ Perceived.stress + CESD.10 + GAD.7

    # regressions
    PAQ_Avoidance_scaled ~ Agreeableness2M + ConscientiousnessM + ExtraversionM +
                              NeuroticismM + OpennessM +
                              CAT_fearfulness + CAT_human_aggression +
                              CAT_activity_playfulness + CAT_cat_sociability +
```

```
                              CAT_human_sociability + CAT_litterbox_issues +
                              CAT_excessive_grooming +
                              owner_gender + owner_age_num +
                              children + Positive_well + Negative_well
    PAQ_Anxiety_scaled ~ Agreeableness2M + ConscientiousnessM + ExtraversionM +
                              NeuroticismM +
                              CAT_fearfulness + CAT_human_aggression +
                              CAT_activity_playfulness + CAT_cat_sociability +
                              CAT_human_sociability + CAT_litterbox_issues +
                              CAT_excessive_grooming +
                              owner_gender + owner_age_num +
                              children + Positive_well + Negative_well
    Negative_well ~ NeuroticismM + ExtraversionM
    Positive_well ~ NeuroticismM + ExtraversionM
    NeuroticismM ~ owner_gender + owner_age_num
    Agreeableness2M ~ owner_gender + owner_age_num
    ConscientiousnessM ~ owner_age_num

    # correlations
    Negative_well ~~ owner_gender
    Positive_well ~~ owner_gender
    PAQ_Avoidance_scaled ~~ 0*PAQ_Anxiety_scaled
    NeuroticismM ~~ ConscientiousnessM
    NeuroticismM ~~ ExtraversionM
    OpennessM ~~ ExtraversionM
    ExtraversionM ~~ ConscientiousnessM
    Agreeableness2M ~~ NeuroticismM + OpennessM
    CAT_cat_sociability ~~ CAT_activity_playfulness + CAT_human_aggression +
CAT_litterbox_issues
    CAT_human_sociability ~~ CAT_activity_playfulness + CAT_fearfulness
    CAT_human_aggression ~~ CAT_fearfulness
    CAT_litterbox_issues ~~ CAT_excessive_grooming
'

fit.cat.sem.5 <- sem(cats.sem.5, estimator="MLR", missing="ml.x", fixed.x=T, data =
paq.cats)
posinegawell.scale.large.cat <- fitmeasures(fit.cat.sem.5, c("cfi","tli","rmsea","srmr"))




#### **** Cat model 6: Positive & negative wellbeing, latent paq, large personality ####

cats.sem.6 <- '
    # latent variables
    Positive_well =~ Life.satisfaction + Wellbeing
    Negative_well =~ Perceived.stress + CESD.10 + GAD.7
    Anxiety =~ Anx1 + Anx2 + Anx3 + Anx4 + Anx5 + Anx6 + Anx7 + Anx8 + Anx9 + Anx10 +
Anx11 + Anx12 + Anx13
    Avoidance =~ Avoid1 + Avoid2 + Avoid3 + Avoid4 + Avoid5 + Avoid6 + Avoid7 + Avoid8 +
                    Avoid9 + Avoid10 + Avoid11 + Avoid12 + Avoid13

    # regressions
    Avoidance ~ Agreeableness2M + ConscientiousnessM + ExtraversionM +
                              NeuroticismM + OpennessM +
```

```
                              CAT_fearfulness + CAT_human_aggression +
                              CAT_activity_playfulness + CAT_cat_sociability +
                              CAT_human_sociability + CAT_litterbox_issues +
                              CAT_excessive_grooming +
                              owner_gender + owner_age_num +
                              children + Positive_well + Negative_well
    Anxiety ~ Agreeableness2M + ConscientiousnessM + ExtraversionM +
                              NeuroticismM +
                              CAT_fearfulness + CAT_human_aggression +
                              CAT_activity_playfulness + CAT_cat_sociability +
                              CAT_human_sociability + CAT_litterbox_issues +
                              CAT_excessive_grooming +
                              owner_gender + owner_age_num +
                              children + Positive_well + Negative_well
    Positive_well ~ NeuroticismM + ExtraversionM
    Negative_well ~ NeuroticismM + ExtraversionM
    NeuroticismM ~ owner_gender + owner_age_num
    Agreeableness2M ~ owner_gender + owner_age_num
    ConscientiousnessM ~ owner_age_num

    # correlations
    Positive_well ~~ owner_gender
    Negative_well ~~ owner_gender
    Avoidance ~~ 0*Anxiety
    NeuroticismM ~~ ConscientiousnessM
    NeuroticismM ~~ ExtraversionM
    OpennessM ~~ ExtraversionM
    ExtraversionM ~~ ConscientiousnessM
    Agreeableness2M ~~ NeuroticismM + OpennessM
    CAT_cat_sociability ~~ CAT_activity_playfulness + CAT_human_aggression +
CAT_litterbox_issues
    CAT_human_sociability ~~ CAT_activity_playfulness + CAT_fearfulness
    CAT_human_aggression ~~ CAT_fearfulness
    CAT_litterbox_issues ~~ CAT_excessive_grooming
'

fit.cat.sem.6 <- sem(cats.sem.6, estimator="MLR", missing="ml.x", fixed.x=T, data =
paq.cats)
posinegawell.latent.large.cat <- fitmeasures(fit.cat.sem.6, c("cfi","tli","rmsea","srmr"))




#### **** Cat model 7: Positive & negative wellbeing, scales, small personality ####


cats.sem.7 <- '
    # latent variables
    Positive_well =~ Life.satisfaction + Wellbeing
    Negative_well =~ Perceived.stress + CESD.10 + GAD.7

    # regressions
    PAQ_Avoidance_scaled ~ ConscientiousnessM + ExtraversionM +
                              OpennessM +
```

```
                              CAT_fearfulness + CAT_human_aggression +
                              CAT_activity_playfulness + CAT_cat_sociability +
                              CAT_human_sociability + CAT_litterbox_issues +
                              CAT_excessive_grooming +
                              owner_gender + owner_age_num +
                              children + Positive_well + Negative_well
    PAQ_Anxiety_scaled ~ NeuroticismM +
                              CAT_fearfulness + CAT_human_aggression +
                              CAT_activity_playfulness + CAT_cat_sociability +
                              CAT_human_sociability + CAT_litterbox_issues +
                              CAT_excessive_grooming +
                              owner_gender + owner_age_num +
                              children + Positive_well + Negative_well
    Positive_well ~ NeuroticismM + ExtraversionM
    Negative_well ~ NeuroticismM + ExtraversionM
    NeuroticismM ~ owner_gender + owner_age_num
    ConscientiousnessM ~ owner_age_num

    # correlations
    Positive_well ~~ owner_gender
    Negative_well ~~ owner_gender
    PAQ_Avoidance_scaled ~~ 0*PAQ_Anxiety_scaled
    NeuroticismM ~~ ConscientiousnessM
    NeuroticismM ~~ ExtraversionM
    OpennessM ~~ ExtraversionM
    ExtraversionM ~~ ConscientiousnessM
    CAT_cat_sociability ~~ CAT_activity_playfulness + CAT_human_aggression +
CAT_litterbox_issues
    CAT_human_sociability ~~ CAT_activity_playfulness + CAT_fearfulness
    CAT_human_aggression ~~ CAT_fearfulness
    CAT_litterbox_issues ~~ CAT_excessive_grooming
'

fit.cat.sem.7 <- sem(cats.sem.7, estimator="MLR", missing="ml.x", fixed.x=T, data =
paq.cats)
posinegawell.scale.small.cat <- fitmeasures(fit.cat.sem.7, c("cfi","tli","rmsea","srmr"))



#### **** Cat model 8: Positive & negative wellbeing, latent paq, small personality ####


cats.sem.8 <- '
    # latent variables
    Positive_well =~ Life.satisfaction + Wellbeing
    Negative_well =~ Perceived.stress + CESD.10 + GAD.7
    Anxiety =~ Anx1 + Anx2 + Anx3 + Anx4 + Anx5 + Anx6 + Anx7 + Anx8 + Anx9 + Anx10 +
Anx11 + Anx12 + Anx13
    Avoidance =~ Avoid1 + Avoid2 + Avoid3 + Avoid4 + Avoid5 + Avoid6 + Avoid7 + Avoid8 +
                    Avoid9 + Avoid10 + Avoid11 + Avoid12 + Avoid13

    # regressions
    Avoidance ~ ConscientiousnessM + ExtraversionM +
                              OpennessM +
```

```
                              CAT_fearfulness + CAT_human_aggression +
                              CAT_activity_playfulness + CAT_cat_sociability +
                              CAT_human_sociability + CAT_litterbox_issues +
                              CAT_excessive_grooming +
                              owner_gender + owner_age_num +
                              children + Positive_well + Negative_well
    Anxiety ~                 NeuroticismM +
                              CAT_fearfulness + CAT_human_aggression +
                              CAT_activity_playfulness + CAT_cat_sociability +
                              CAT_human_sociability + CAT_litterbox_issues +
                              CAT_excessive_grooming +
                              owner_gender + owner_age_num +
                              children + Positive_well + Negative_well
    Positive_well ~ NeuroticismM + ExtraversionM
    Negative_well ~ NeuroticismM + ExtraversionM
    NeuroticismM ~ owner_gender + owner_age_num
    ConscientiousnessM ~ owner_age_num

    # correlations
    Positive_well ~~ owner_gender
    Negative_well ~~ owner_gender
    Avoidance ~~ 0*Anxiety
    NeuroticismM ~~ ConscientiousnessM
    NeuroticismM ~~ ExtraversionM
    OpennessM ~~ ExtraversionM
    ExtraversionM ~~ ConscientiousnessM
    CAT_cat_sociability ~~ CAT_activity_playfulness + CAT_human_aggression +
CAT_litterbox_issues
    CAT_human_sociability ~~ CAT_activity_playfulness + CAT_fearfulness
    CAT_human_aggression ~~ CAT_fearfulness
    CAT_litterbox_issues ~~ CAT_excessive_grooming
'

fit.cat.sem.8 <- sem(cats.sem.8, estimator="MLR", missing="ml.x", fixed.x=T, data =
paq.cats)
posinegawell.latent.small.catt <- fitmeasures(fit.cat.sem.8,
c("cfi","tli","rmsea","srmr"))




#### ** Model fit comparison ####

rbind(totwell.scale.large.cat,totwell.latent.large.cat,
      totwell.scale.small.cat,totwell.latent.small.cat,
      posinegawell.scale.large.cat,posinegawell.latent.large.cat,
      posinegawell.scale.small.cat,posinegawell.latent.small.cat)

# cfi: 1&3 equal, 5&7 next but far behind
# tli: 1&3 equal
# rmsea: 1 best, then 3, then 2
# srmr: 3 best, then 1

### FIRST OVERALL BEST
```

```r
#### ** Final cat model ####

## first model


cats.sem.final <- '
    # latent variables
    Total_wellbeing =~ Life.satisfaction + Perceived.stress + Wellbeing +
                CESD.10 + GAD.7

    # regressions
    PAQ_Avoidance_scaled ~ Agreeableness2M + ConscientiousnessM + ExtraversionM +
                          NeuroticismM + OpennessM +
                          CAT_fearfulness + CAT_human_aggression +
                          CAT_activity_playfulness + CAT_cat_sociability +
                          CAT_human_sociability + CAT_litterbox_issues +
                          CAT_excessive_grooming +
                          owner_gender + owner_age_num +
                          children + Total_wellbeing
    PAQ_Anxiety_scaled ~ Agreeableness2M + ConscientiousnessM + ExtraversionM +
                          NeuroticismM +
                          CAT_fearfulness + CAT_human_aggression +
                          CAT_activity_playfulness + CAT_cat_sociability +
                          CAT_human_sociability + CAT_litterbox_issues +
                          CAT_excessive_grooming +
                          owner_gender + owner_age_num +
                          children + Total_wellbeing
    Total_wellbeing ~ NeuroticismM + ExtraversionM
    NeuroticismM ~ owner_gender + owner_age_num
    Agreeableness2M ~ owner_gender + owner_age_num
    ConscientiousnessM ~ owner_age_num

    # correlations
    Total_wellbeing ~~ owner_gender
    PAQ_Avoidance_scaled ~~ 0*PAQ_Anxiety_scaled
    NeuroticismM ~~ ConscientiousnessM
    NeuroticismM ~~ ExtraversionM
    OpennessM ~~ ExtraversionM
    ExtraversionM ~~ ConscientiousnessM
    Agreeableness2M ~~ NeuroticismM + OpennessM
    CAT_cat_sociability ~~ CAT_activity_playfulness + CAT_human_aggression +
CAT_litterbox_issues
    CAT_human_sociability ~~ CAT_activity_playfulness + CAT_fearfulness
    CAT_human_aggression ~~ CAT_fearfulness
    CAT_litterbox_issues ~~ CAT_excessive_grooming
'

# run the model
fit.cat.sem.final <- sem(cats.sem.final, estimator="MLR", missing="ml.x", fixed.x=T, data
= paq.cats)

# extract model fit measures
```

```
final.cat.model <- fitmeasures(fit.cat.sem.final, c("cfi","tli","rmsea","srmr"))

# standardized and unstandardized estimates
standardizedSolution(fit.cat.sem.final,output="pretty")
parameterEstimates(fit.cat.sem.final)


#### ** RMSEA of null model ####

nullRMSEA(fit.cat.sem.final)
```
